# International consensus classification of early tuberculosis states to guide research for improved care and prevention: A Delphi exercise

**DOI:** 10.1016/S2213-2600(24)00028-6

**Published:** 2024-03-22

**Authors:** Anna K Coussens, Syed MA Zaidi, Brian A Allwood, Puneet K Dewan, Glenda Gray, Mikashmi Kohli, Tamara Kredo, Ben J Marais, Guy Marks, Leo Martinez, Morten Ruhwald, Thomas J Scriba, James A Seddon, Phumeza Tisile, Digby F Warner, Robert J Wilkinson, Hanif Esmail, Rein MGJ Houben

**Affiliations:** 1 Infectious Diseases and Immune Defence Division, The Walter and Eliza Hall Institute (WEHI), Australia; 2 Centre for Infectious Diseases Research in Africa, Institute of Infectious Disease and Molecular Medicine, University of Cape Town, South Africa; 3 Department of Medical Biology, University of Melbourne, Australia; 4 WHO Collaborating Centre on Tuberculosis Research and Innovation, Institute for Global Health, University College London, London, UK; 5 MRC Clinical Trials Unit at University College London, London, UK; 6 Department of Public Health, National University of Medical Sciences, Pakistan; 7 Department of Pulmonology, University of Stellenbosch, Stellenbosch, South Africa; 8 Tuberculosis & HIV, Bill and Melinda Gates Foundation, Seattle, USA; 9 Health Systems Research Unit, South Africa Medical Research Council, Cape Town, South Africa; 10 Health Programmes, FIND, Geneva, Switzerland; 11 Sydney Infectious Diseases Institute, University of Sydney, Sydney, Australia; 12 WHO Collaborating Centre in Tuberculosis, University of Sydney, Sydney, Australia; 13 Department of Clinical Medicine, Faculty of Medicine and Health, University of Sydney, Sydney, Australia; 14 Department of Epidemiology, Boston University School of Public Health, Boston, United States; 15 South African Tuberculosis Vaccine Initiative, Institute of Infectious Disease and Molecular Medicine, University of Cape Town, Cape Town, South Africa; 16 Department of Infectious Disease, Imperial College London, London, United Kingdom; 17 Desmond Tutu TB Centre, Department of Paediatrics and Child Health, Stellenbosch University, South Africa; 18 TB PROOF, South Africa; 19 Department of Pathology, University of Cape Town, Cape Town, South Africa; 20 Department of Infectious Diseases, Imperial College London, London, UK; 21 The Francis Crick Institute, London, UK; 22 TB Modelling Group, TB Centre, London School of Hygiene and Tropical Medicine, London, UK; 23 Department of Infectious Disease Epidemiology, London School of Hygiene and Tropical Medicine, London, UK

## Abstract

The current active/latent paradigm of tuberculosis (TB) largely neglects the documented spectrum of disease. Lack of consistency on definitions, terminology and diagnostic criteria for different TB states constrains progress in research and product development required to achieve TB elimination.

We reached consensus on a set of conceptual states, related terminology and research gaps through a Delphi process, involving 64 experts, representing a wide range of disciplines, sectors, income settings and geographies.

The resulting International Consensus for Early TB (ICE-TB) framework distinguishes disease from infection by the presence of macroscopic pathology and defines two subclinical and two clinical tuberculosis states based on reported symptoms or signs of TB, further differentiated by likely infectiousness. Presence of viable *M. tuberculosis* and an associated host response are prerequisites for all infection and disease states.

Our framework provides a clear direction for TB research, which in time with scientific progress, will improve TB clinical care and elimination policies.

## Introduction

Tuberculosis (TB) has been a major cause of morbidity and mortality for thousands of years. [1] Despite the availability of a vaccine for over 100 years and drugs for over 75 years, TB remains the leading cause of death globally from an infectious disease. [2,3] TB has a complex natural history and pathogenesis which is still incompletely understood. A minority of people exposed and considered infected with *Mycobacterium tuberculosis* (*Mtb*) progress to disease. The incubation period is variable, with highly heterogeneous disease presentation and progression pathways. Faced with this complexity, a simplifying framework has value in helping to guide and communicate the public health approach, management, and scientific investigation of TB. Periodically, the nature of the framework inevitably must evolve reflecting new scientific understanding, tools for diagnosis and intervention, and public health priorities.

The development of curative antibiotic treatment from the late 1940s resulted in a radical change in the approach to managing TB and led to a shift in approach to disease classification. Prior to this period, TB was recognised as a dynamic process, [4] with prognostic stratification by disease extent on chest X-ray (CXR) and the precise classification of current disease state (arrested, quiescent, active, etc.). [5] This approach was abandoned and in its place, a simple binary paradigm of latent infection and active disease emerged. [6,7] The diagnosis of disease increasingly focused on microbiological confirmation as a prerequisite for receiving curative treatment. Infection was inferred from evidence of immune sensitisation to *Mtb* antigens in the absence of features of disease, with therapy recommended in certain situations to prevent progression to disease. [6] This binomial simplicity facilitated the development and improvement of programmatic management of TB during the 1990s particularly with the DOTS strategy, which emphasised case detection and standardised treatment of symptomatic people with sputum smear-positive pulmonary TB. [8] While this approach prevented millions of TB deaths [9,10], it had lesser impact on transmission and disease incidence, possibly because millions of individuals with TB do not present to health facilities or receive care.

The globally adopted EndTB strategy aims to dramatically reduce TB incidence and mortality by 2030, with ultimate TB elimination. [11–13] With these ambitious goals, which are currently not on track to being met, the limitations of the binary active disease vs latent infection paradigm are increasingly apparent and can hinder progress. Since 2000, over 25 national TB prevalence surveys in Asia and Africa, conducted in over 2,000,000 people, have shown that approximately 50% of people with sputum culture-positive and potentially infectious TB in the community do not report TB symptoms, as traditionally defined. [14] Natural history descriptions suggest that individuals could remain in this asymptomatic or minimally-symptomatic state for several months or years. [15,16] This group is not identified by current symptom-based active case finding strategies. Furthermore, although immunocompetent adults with *Mtb* infection that subsequently progress to pulmonary disease contribute to transmission, attempts to implement TB preventive treatment (TPT) for this group as a public health strategy to interrupt transmission have faced operational challenges [17] This is due to the low predictive value of diagnostic tests for infection to identify those at highest risk of disease progression, resulting in over 100 people requiring TPT to prevent a single occurrence of disease in some settings. [18] Tests to identify more precisely those who stand to benefit most from TPT are a priority, but the current binary framework does not provide a satisfactory mechanism to approach this.

These limitations have been well documented. [19,20] In a scoping review we performed to inform this work, we identified 40 articles proposing that TB infection and disease is better represented as multiple states beyond just latent and active. [21] However, there was a lack of consistency in conceptual and diagnostic definitions, in the number and type of additional TB states described, and in their terminology. This has understandably led to confusion among researchers, practitioners and policymakers on the precise meaning and definitions of terms related to early disease states that precede symptomatic TB.

An updated classification for TB that accommodates key disease states but retains sufficient simplicity to support pragmatic research and implementation is needed. Such a framework should provide clarity and consensus for researchers on the definition of distinct conceptual states, consistent terminology and identify research gaps but also be able to facilitate communication of the public health approach to TB and ultimately improve individual patient management. To address this need, an International Consensus Group for Early TB was convened, comprising a multidisciplinary group of TB survivors, academics, policymakers, clinicians, industry and funders. In this paper we aim to report on the process and outcomes of this consensus process that resulted in the International Consensus for Early TB (ICE-TB) classification framework.

## Methods

**Figure 1:**
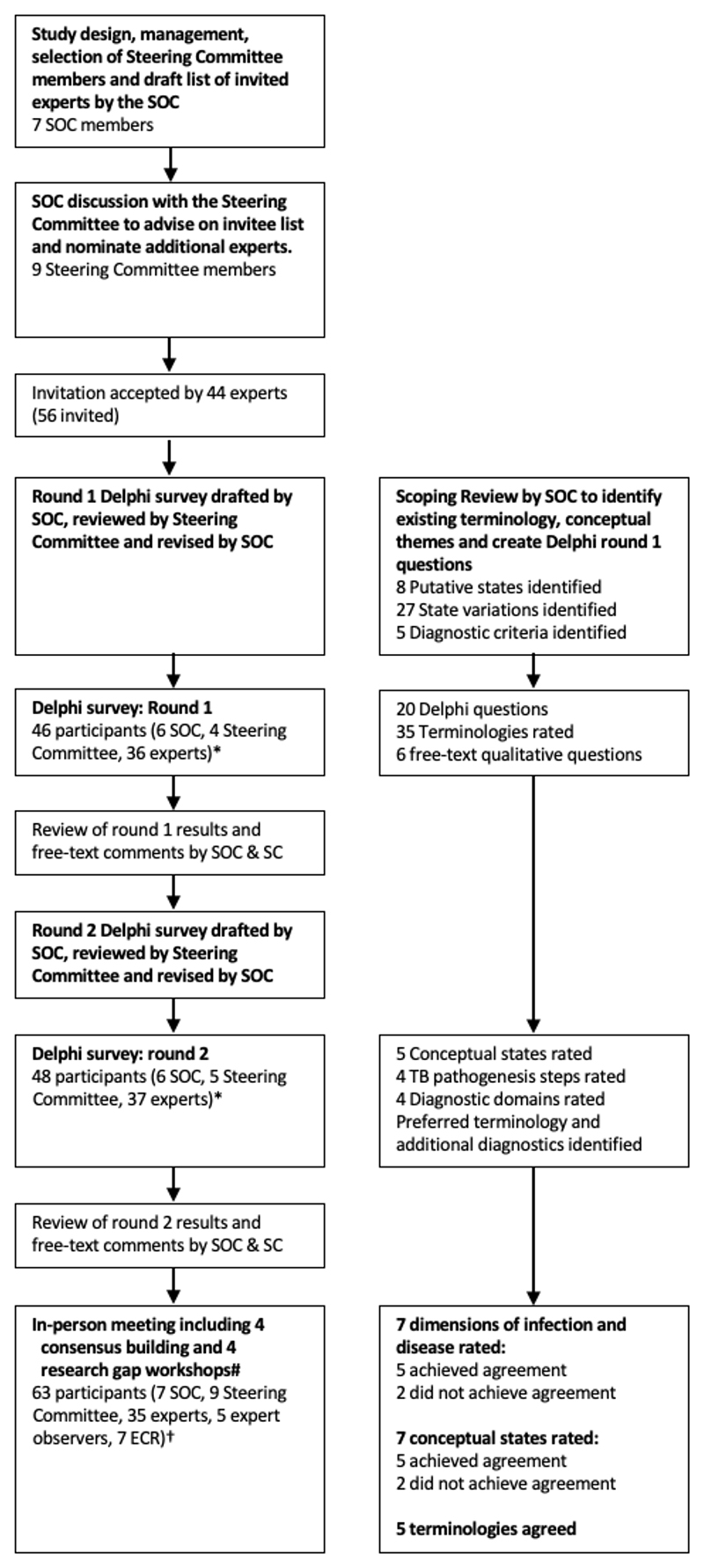
a-b: Meeting process (a) and delegate overview (b)

We took a systematic Delphi process towards developing consensus through a three-step approach (Figure 1 and Appendix page 2). [22,23] In the first step, a scoping review was conducted through a systematic search without date restrictions, for review articles describing TB as a multi-state disease. [21] The results of the scoping review provided the evidence-base for the second step which included two rounds of expert surveys between November 2022 and January 2023. Building on the survey results, the third step consisted of an in-person symposium in February 2023 during which the final consensus on conceptual states and terminologies was reached.

### Participants

The project was conceived by AC, HE and RH who formed a Scientific Organising Committee (SOC) to conduct and manage the project, inviting scientists and clinicians with long-standing interest in early states of tuberculosis (JS, DW, SZ) and a clinician and policy expert with expertise in consensus methodology (TK). The SOC invited experts in this area to compose a steering committee (SC) including senior academics, funders (NIH and Bill and Melinda Gates Foundation) and representatives from WHO, the International Union against TB and Lung Disease, FIND (diagnostic product development organisation) and TB Proof (advocacy group) (PD, PF, GG, NI, MK, GM, MR, PT, RW).

### Participants

To enable constructive in-person discussions, participation in the consensus process was limited to 64 expert delegates (of which 56 attended the in-person symposium), supported by seven Early Career Researchers (ECR) invited from local universities through an open call to act as observers and support the in-person meeting through a rapporteur role (Appendix page 4). Expert delegates were purposefully selected to ensure a diverse and broadly representative group able to provide opinions that were relevant to a wide spectrum of stakeholders and reflected a range of priorities, geographical locations with balance in income settings, gender, professional disciplines as well as lived and working experiences (Table 1 and Appendix page 4). A list of potential participants was drafted by the SOC, with further input by the SC, representing academics drawn from a range of scientific specialties, as well as clinicians (adult, paediatric and public health), policymakers, TB programme and governmental representatives, TB advocates and survivors, and funders. Invitations were sent to 56 experts of which 44 accepted (79%), and 3 subsequently withdrew. Eight invited experts who completed the Delphi Process could not attend the in-person meeting and were replaced with two experts from the waiting list (1 academic clinical practice, 1 policy) and five expert observers (4 funder and 1 industry), ensuring maintenance of gender and geographical representation.

### Delphi process - online surveys

The results of the scoping review helped inform questions for the expert surveys. This included commonly used terminologies, definitions, nomenclature and diagnostic criteria. These were utilised to derive the questions and responses for the surveys, including specifics related to conceptual and diagnostic definitions for each state. The surveys were drafted by the SOC and reviewed by the SC. A mix of semi-quantitative, open and closed ended questions were utilised. Pilot testing of the survey was conducted with respondents consisting of TB researchers from the MRC Clinical Trials Unit at UCL and feedback was obtained regarding the clarity and relevance for each question. Modifications were made to the surveys based on the feedback provided and were finalized by the SOC. Both surveys were subsequently developed on Mentimeter in English and distributed to the participants through an online link via email. Two authors (SMAZ and HE) analysed the survey results using MS Excel.

The first survey included 20 Delphi and six free-text qualitative questions that explored the perceived need for a novel framework, perspectives on TB states, natural history of TB (including the dimensions that define disease) and research priorities. Participants also rated 35 terminologies for TB states identified from the scoping review. The first survey identified broad agreement among respondents on the need for a novel classification for TB states and on key steps in the disease pathogenesis. Participants frequently described the current binary classification as an *“over-simplification”*. Important distinguishing criteria for TB states were also identified. “Transmission potential / infectiousness” and “ability to discriminate using current or future diagnostics” were identified as key criteria for distinguishing states whereas “*pathological damage”* was most frequently mentioned as the starting point for disease.

Results from the first survey were reviewed by both committees and helped derive questions for the second survey. Free text responses were analysed qualitatively, and common phrases and themes were identified to supplement the Delphi questions. At the beginning of the second survey, summary results from the first survey were shared with participants online using Mentimeter slides. This was followed by questions asking participants to rate the relevance of four key steps in TB pathogenesis, five proposed conceptual TB states and four diagnostic criteria. Participants broadly agreed on the steps in pathogenesis, however, there was disagreement on diagnostic criteria and relevance for some of the proposed states. Novel diagnostic tests that are not in routine clinical use but are potentially applicable for differentiating between disease states were also identified.

Results from both online surveys (see Appendix page 15) were reviewed by the SOC and the SC and key discussion points were identified. They were subsequently presented to all participants at the first session of the in-person consensus meeting with the aim of establishing broad areas of agreement and to help focus discussions on areas of disagreement. Word clouds for free text responses generated via Mentimeter were utilised to corroborate findings of the quantitative results by highlighting the most frequently used phrases and themes to the participants.

### Delphi process - in-person consensus meeting

The consensus meeting consisted of plenary sessions where presentations were followed by open discussions chaired by content specialists and moderated by an impartial expert methodologist (TK). In addition, eight small-group workshops were conducted on specific topics of interest, identified from the first round of online Delphi and discussions between the SC and SOC to identify likely areas where identifying a consensus might be challenging. Four expert delegates (BA, BJM, LM, TJS) were invited by the SOC to help design and co-chair each workshop. (See Appendix page 32 for the full meeting agenda, including workshop chairs and participants)

At the conclusion of the symposium, agreement on all stages and definitions for the proposed novel framework was reached through informal polling and discussion to reach a broad consensus. without the need for a formal vote on any specific disagreement. Several expert delegates (n=10) declared *a priori* that they would not vote, including the invited observers, although this was not needed given the consensus reached without need for formal vote. Further details on the in-person symposium consensus process are provided in Appendix page 2-14. Following on from the in-person consensus meeting this position paper was written by the SOC with input from the SC and workshop co-chairs. All members of the ICE-TB group were sent a draft of the paper and invited to online-meetings for further involvement to ensure the draft text, tables and diagrams accurately represented discussions.

## Results

### Scoping review

The scoping review identified considerable inconsistency in terminology and definitions used to describe conceptual disease and non-disease states of Mtb infection, with a total of twenty-seven distinct state variations identified. As previously described,[21] these 27 variations could be subdivided into eight putative states centred around common concepts with suggested nomenclature and diagnostic criteria. These eight putative states and 35 selected terminologies were utilised to develop the Delphi survey questions, including specifics related to conceptual and diagnostic definitions, and perceived usefulness of each terminology.

### Demographics and characteristics of Delphi participants

The first round of the Delphi survey was conducted between November 17-30, 2022, and the second round between January 13-30, 2023. 51 of 54 individuals (94%, 25 Academic, 16 Clinical Academic/Clinical Practice, 2 Patient perspective/Lived experience, 6 Policy, 2 Funder) who initially agreed to participate in the survey completed questions in at least one round (6 of 60 who accepted invitations were observers for the survey [1 SOC, 3 SC, 2 experts]) (Table 1, Figure 1). A total 46 participants responded to the first survey and 48 responded to the second. The three individuals who completed neither round withdrew from participation due to scheduling conflicts, at varying stages after acceptance. Delphi survey participants had broad representation, including 39% female, 61% male, nationality 51% from LMIC, 49% from HIC, 20% from Africa, 22% from the Americas, 24% from Europe, 4% from Eastern Mediterranean, 18% from South-East Asia and 14% from Western Pacific.

The in-person meeting was held February 1-2, 2023, with 63 participants (28 Academic, 16 Clinical Academic/Clinical Practice, 3 Patient perspective/Lived experience, 7 Policy, 8 Funder, 1 industry), including 43/51 (84%) Delphi survey participants, 7 new invited experts (replacing 8 who completed the Delphi that could not attend due to visa delays (6) or scheduling conflict (2)) and 7 local ECR (4 Academic, 3 Clinical Academic/Clinical Practice). Gender and nationality representation was similar to the Delphi survey with geographical representation 35% from Africa, 12% from the Americas, 25% from Europe, 3% from Eastern Mediterranean, 8% from South-East Asia and 8% from Western Pacific (Table 1).

### Principles for defining new infection and disease states

Results of the Delphi surveys are detailed in Appendix page 15. Round 1 results established agreement within the group that the current active/latent TB binary paradigm was insufficient both for research (89%) or programmatic purposes (76%) to achieve TB elimination and that a new framework representing more TB states would be beneficial (76%). There was also agreement that each conceptual state considered should have the possibility of targeted intervention to provide defined benefits to either the individual (through prevention and care that improves health and wellbeing) or population/society (through strategies to reduce transmission).

Based on the survey results presented at the in-person meeting we then agreed on a set of principles for the framework. First, a new framework should be parsimonious, i.e. include only as many states as needed and avoid unnecessary subcategories. Second, it should be internally consistent, by applying the same criteria throughout. Third, as over a quarter of TB either occurs in children or is exclusively extrapulmonary [17], a new framework should conceptually cover all presentations of TB and not be restricted to adult pulmonary TB.

Building on these principles, we agreed that each conceptual state should reflect pathophysiological processes, rather than be bound solely by practical considerations, such as the ability to identify them with existing diagnostic tools. We also we agreed a conceptual state should reflect the current TB state of the host, and not be predicated on an anticipated future trajectory, which may or may not happen.

Finally, there was consensus regarding the non-linear trajectory of TB, recognising that once the disease process starts, people may fluctuate between infectious and non-infectious states, and between the presence and absence of specific symptoms. Over time, an individual’s disease may ultimately resolve or not. [22] The concept of non-linearity across the spectrum of TB was widely accepted in the early 20^th^ century [4,5] and has been borne out by recent reviews of historical and contemporary data [15,23] and longitudinal studies of asymptomatic TB contacts using high-resolution imaging. [24]

### Dimensions of infection and disease states

During the first online Delphi survey, we explored views on the points along the natural history of TB that should be considered as disease. “The point at which inflammatory/infiltrative pathology to *Mtb* is evident through imaging” was the earliest point where the majority agreed or strongly agreed should be considered disease, whereas the majority disagreed or strongly disagreed that “the point at which a granuloma is formed containing replicating *Mtb*” should be considered disease. The reasoning for this related to recognition of the significance of tissue damage and the potential for impact on organ structure or function being a distinguishing feature of disease (Appendix page 15).

Within the Delphi surveys, we also asked delegates to indicate the dimensions which could define TB states, seven reaching majority selection: the presence of 1) viable *Mtb*, 2) host response, 3) macroscopic pathology, 4) infectiousness, 5) TB symptoms and signs, 6) potential for progression and 7) treatment approach.

During in-person Symposium discussions, two features were excluded; potential for progression (given consensus to base conceptual definitions on current not future states) and treatment approach (which will continually evolve). The remaining features were considered fundamental pathophysiological components.

Of these five pathophysiologic features, it was agreed that there are two prerequisite dimensions for all states of infection and disease, 1) the presence of viable *Mtb* and 2) an associated host response (see Table 2 for definitions). Whilst both vary qualitatively between states, they do not currently help to distinguish between states. The prerequisite of both distinguishes all states from the concept of colonisation where bacteria are present on or within the host in the absence of a host response; whether such a state exists for *Mtb* remains to be determined. It also emphasises that T cell *Mtb* antigen immunoreactivity alone (as determined by Tuberculin Skin Test [TST] or Interferon Gamma Release Assays [IGRA]) is insufficient to define current infection, as T cell memory can persist following *Mtb* clearance [25]. In addition, these tests also do not detect all memory responses to Mtb and can also be potentially falsely negative as Mtb antibodies can be present in individuals who are not immunoreactive by TST or IGRA.

**Figure 2:**
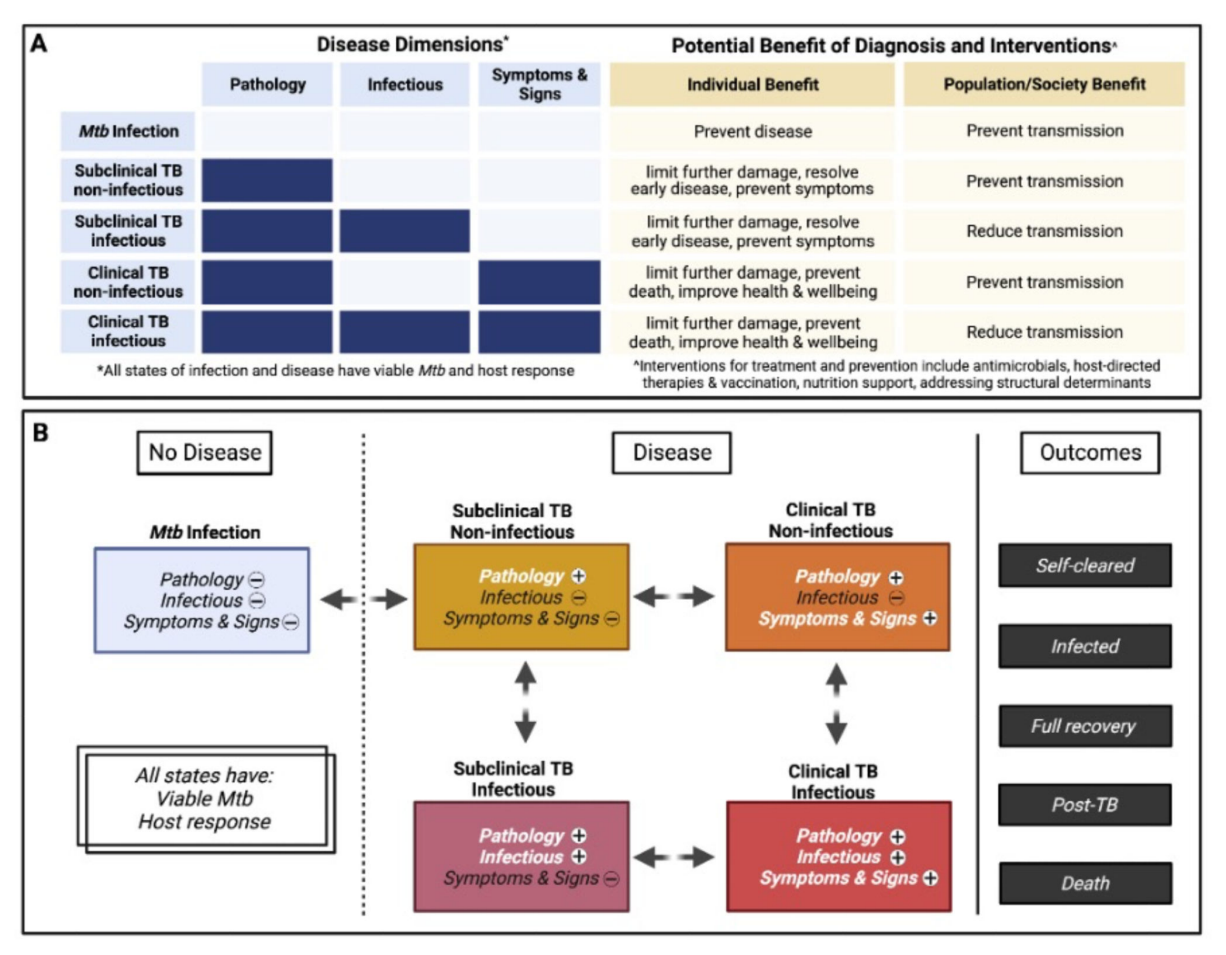
a-b: Overview of Delphi consensus process and participantsFigure 2a-b: Conceptual Mtb infection and TB states identified with consideration of benefit resulting from diagnosis and treatment (2a) and pathways across infection and disease states (2b). Pathology = macroscopic pathology, Infectious = ability to cause new Mtb infections, Symptoms & Signs = TB symptoms and signs, Self-cleared = absence of viable Mtb after Mtb infection, never crossed disease threshold and not received treatment, Infected = persistence of Mtb infection, including after antibiotic or self cure from disease, remains at risk of developing disease. Full recovery = Both disease and infection fully resolved without post-TB impairment, with or without treatment, Post-TB = disease or disability due to damage caused by TB pathology after microbiological or self-cure. Figure created with Biorender.com

Having defined two prerequisite dimension of all states, the remaining three pathophysiological components were agreed as distinct disease dimensions: 1) presence of macroscopic TB pathology, 2) infectiousness of the host, and 3) TB symptoms or signs (see Table 2 for definitions). These three dimensions, presenting in distinct combinations, define four different states of TB (Figure 2a-b, and Table 3).

#### Macroscopic pathology

Macroscopic pathology was agreed as the first disease distinguishing dimension with its presence a prerequisite for all disease states, potential infectiousness (when occurring in the respiratory tract) and symptoms and signs of TB.

Macroscopic pathology here is distinct from a contained granuloma or completely healed lesions, referring to the cellular infiltration occurring after failure of *Mtb* containment. This may be observed directly on anatomical samples (biopsy or autopsy), by clinical examination, or by imaging. It was recognised that the very initial stages of this infiltration may be microscopic and below the limit of detection of current imaging approaches (i.e. 0.25mm with ultra-high resolution CT). However, this microscopic pathology would not impact organ structure or function and it is appropriate to place the disease threshold as macroscopic pathology, which also facilitates detection.

Treatment in those with macroscopic pathology could minimise the unfavourable consequences for the individual associated with pathological tissue damage and chronic inflammation as a part of a person-centred approach to management of TB.

#### Infectiousness

The second dimension, infectiousness, reflects the ability of an individual to aerosolise or expectorate *Mtb* from the respiratory tract which has the potential to cause new *Mtb* infections, driving the societal impact of TB through *Mtb* transmission. Macroscopic pathology will be present in the lung or respiratory tract. Establishing infectiousness at an individual level remains challenging and there is considerable heterogeneity in the degree of infectiousness between though able to aerosolise or expectorate *Mtb* which is poorly understood. However, conceptually it is a key point of intervention as reducing or, where possible, preventing transmission from infectious individuals is one of the main goals of TB programmes along with improving individual outcomes.

#### Symptoms and signs of TB

Clinical characteristics represent the third dimension. Present with or without infectiousness, symptoms and signs are caused by the host response to *Mtb* and may prompt the individual to seek care, enabling low-cost passive case detection which has been the cornerstone of TB care for decades, as well as a potential starting point for clinic and community-based screening programmes. [26]

### International Consensus for Early TB (ICE-TB) framework

Guided by the consensus principles and considerations, we derived a framework with five conceptual states (1 non-disease and 4 disease) and consistent terminology (see Appendix page 30).

#### Mtb infection

Conceptually a non-disease state, where viable *Mtb* exist in the host but are effectively contained by the immune response. The individual has no macroscopic pathology or symptoms or signs consistent with TB and is non-infectious.

Whilst *Mtb* infection must precede the onset of disease, this state is also the most problematic to diagnose with current tools, such as the TST or IGRA which only detect immunoreactivity to *Mtb* (see below). [27] A minority of those infected (currently only inferred by conversion to positive immunoreactivity following exposure) will develop disease. [28,29] While *Mtb* infection was assumed to be lifelong in the classic framework, evidence now suggests that a substantial proportion of individuals self-clear infection. [30,31] Existing estimates for the proportion of people with *Mtb* infection are based on immunoreactivity, [32] likely leading to an overestimate.

Successful antibiotic or immune-mediated clearance of viable bacteria, or maintaining granuloma control, would decrease risk of disease development and negative outcomes arising. Similarly, potential transmission in the future would be prevented (Figure 2a, top row). Once an individual fails to contain *Mtb* infection and develops macroscopic pathology the following subclinical and clinical disease states are recognised.

#### Subclinical TB, non-infectious

In this disease state, macroscopic pathology is present, but the individual is not infectious, and symptoms or signs, if present, are not recognised or not acknowledged by the individual, or are insufficient to seek care. Therefore, typically it would be identified through screening using an imaging modality.

Assessing whether the observed pathology is due to viable *Mtb* when microbiological investigation of samples are negative is challenging (see Diagnostic Considerations and Research Priorities below). Subclinical non-infectious TB could occur at extrapulmonary sites but would require a screening mechanism other than chest imaging to be detected.

Treatment of this state has the benefit to the individual of limiting further pathological damage [33] and resolving chronic inflammation, which if left could cause further illness, post-TB sequelae or death and could impact other comorbidities. Treatment also prevents potential future infectiousness.

#### Subclinical TB, infectious

Individuals with subclinical, infectious TB are capable of transmitting *Mtb* with macroscopic pathology present, but symptoms, if present, are not recognised or not acknowledged by the individual, or are insufficient to seek care.

Primarily occurring in pulmonary disease, this state results from progressive immunopathology allowing *Mtb* escape into tissue-air interfaces, where *Mtb* aerosolization or expectoration contributes to transmission, although transmission intensity likely varies over time, with lesion nature and perhaps infecting strain of *Mtb*. [34] Aerosolization can occur without coughing, through breathing and speaking, although it has not yet been established whether *Mtb* released in this method can be cultured, they were confirmed physiologically active. [35,36] Analysis of household contact data has suggested that subclinical TB is infectious, [37,37,38] while modelling analyses of empirical data suggest that individuals can persist in this state for prolonged periods, which means in terms of transmission an average lower bacterial burden could be nevertheless associated with prolonged periods of infectiousness and thereby substantial transmission. [15,39,40]

Contemporary prevalence surveys have shown that this state represents around half of individuals with prevalent infectious pulmonary disease, [14] based on CXR and microbiological testing with culture or PCR-based testing of sputum. In the future, better sampling techniques for respiratory aerosols may further improve the detection of subclinical infectious TB (see below). Although prevalence surveys rarely also identify those positive on sputum microbiology and normal CXR, pathology is typically evident with higher resolution imaging. [24]

Treatment of this state has the benefit of limiting further pathological damage, resolving chronic inflammation, and therefore preventing the potential risk of illness, post-TB sequelae or death. Detection and treatment should also reduce *Mtb* transmission. [41]

#### Clinical TB, non-infectious

This state includes all forms of disease where the affected individual experiences symptoms or signs sufficient for them to be recognised or for the individual to seek care. However, the individual is not infectious.

A substantial proportion of adults presenting clinically with pulmonary TB have bacteriologically-negative sputum, and may be classified in this state. [17] In addition, most extrapulmonary TB in adults and most TB in children falls within this disease state.

Treatment at this stage can arrest pathological damage or promote resolution to improve health and survival for the individual. For pulmonary disease, it can also prevent possible future transmission.

#### Clinical TB, infectious

This state most closely reflects the classic ‘active TB’ (i.e. individuals are infectious based on sputum microbiologically confirmed pulmonary TB, which is diagnosed among individuals experiencing symptoms or signs of TB sufficient for them to be recognised by the individual or prompt them to seek care). Any symptomatic individual able to aerosolise Mtb would be considered to be in this state irrespective of disease at other sites (i.e. they may also have extra-pulmonary disease or disseminated disease)

Contemporary prevalence surveys have shown that this group makes up about half of prevalent pulmonary infectious disease, which contributes strongly to transmission. [14,42,43]

Treatment is key to prevent death from TB, as well as reducing further progressive pathological damage, post-TB sequelae and transmission.

#### Incipient TB

The inclusion of the concept and/or term “incipient” TB was explored in the Delphi process and discussed in person. The consensus was to not include this conceptual state in the framework, since it represented a trajectory rather than a state, which was inconsistent with the consensus principle-based approach (see Appendix page 30 for fuller discussion). In addition, in the on-line Delphi Survey when asked about the use of the term in TB staging on a 5-point Likert scale “incipient TB” itself was less popular than other terms with a mean score of 2.8 (see Appendix page 15).

### Diagnostic considerations

While identifying diagnostic criteria for the states was a desired outcome of the ICE-TB meeting, the lack of validated tools for some states meant this was not feasible. However, acknowledging the limitations, it was agreed that tools across the expanding diagnostic landscape could be used to classify an individuals’ TB state according to the relevant disease dimensions, particularly in a research context. Establishment of appropriate reference standards for the new states and subsequent development of new diagnostic tools will be necessary to define the TB states more accurately. (See Table 3 for a list of potential tools, sample approaches, and likely performance in detecting the disease dimensions).

The issue of imperfect reference standards has long been a challenge for the TB field, especially for extrapulmonary TB, paediatric TB, and TB in people living with HIV, due to the paucibacillary nature of the samples usually available for diagnosis. However, many research groups have designed and validated composite reference standards with predetermined rules, consisting of multiple concurrent or sequential tests and applying statistical methods to correct for the imperfect nature of the existing reference standards, (e.g. for TB meningitis). [44] Additionally, applying Bayesian approaches such as latent class analysis where imperfect reference standards exist, can help minimize misclassification when doing accuracy trials. [45,46]

Following development of a consensus-driven reference standard, it will be imperative to have robust study designs to evaluate diagnostic accuracy and effectiveness of existing and new tools for each TB state. This will require careful considerations around study designs, participant inclusion criteria, statistical approaches, and outcome measures.

#### Viable Mtb

There is currently no validated test of viable *Mtb* that can be used to confirm the state of *Mtb* infection. Validated molecular tools that detect *Mtb* DNA or antigen (i.e. LAM) confirm bacterial antigen presence but not viability, while current host immunoreactivity assays (e.g. TST or IGRA) can only infer recent/previous *Mtb* infection. Repeat tests for immunoreactivity confirming conversion from negative to positive suggests a recent infection event and are associated with an elevated risk of subsequent disease. As test positivity can persist following *Mtb* clearance, the probability of infection varies by exposure timing and frequency. Host response tests to confirm viable *Mtb* infection under diagnostic evaluation include *Mtb*-specific T cell activation markers, which detect T cells actively responding to *Mtb* antigen in the body. [47,48]

#### Host response

Host immunological response can be separated into two types: *Mtb*-specific antigen responses used to monitor infection (as described for Viable *Mtb*) and host-specific responses that reflect ongoing pathophysiological processes. Tests which inform disease processes include blood transcriptional signatures under evaluation for clinical TB diagnosis and progression risk. [49,50] The development of new host response tests for different disease states will be important particularly for non-infectious TB.

#### Macroscopic pathology

Assessment of pathology has been a cornerstone of TB care and research since the development of X-Ray to produce radiographs. CXR remains widely used as a screening and diagnostic tool. Cross sectional imaging with CT considerably increases the sensitivity to identify pathological changes and can be enhanced by FDG-PET, to provide deep insights into the presence of likely TB-associated pathology.[51] However, current radiotracers and imaging approaches are not specific for TB and are suboptimal for monitoring treatment response, which are significant limitations and could result in overtreatment if used alone to guide treatment decisions. There has been progress in the development of PET radiotracers more specific for TB, which could be a valuable research tool. However, this would not have wide implications globally for clinical diagnosis. [52,53] However, development of blood, urine or respiratory biomarkers and diagnostic tests that could detect TB-specific pathology with high sensitivity and specificity could be transformative. [54,55]

#### Infectiousness

Assessment of infectiousness has been based on identifying *Mtb* in respiratory samples, particularly sputum, using sputum smear, culture, or molecular tests. In recent years, new sample modalities are being developed, including upper respiratory tract samples (*e.g*., tongue swabs), and bio-aerosol capture, using the Cough Aerosol Sampling System (CASS) and face mask sampling technologies. [36,56] Identification of *Mtb* in respiratory samples is a key, but only first, step in establishing infectiousness, which is the potential ability to cause new *Mtb* infections (Table 2). Tests ideally need validating through; for example, the guinea pig transmission model [57], measuring *Mtb* infection in household contacts [58,59], or using molecular tools to identify whether epidemiologically linked individuals present with genetically closely linked *Mtb*. [60,61] In this context, the CASS is currently the best-performing tool, showing good correlation with clinical endpoints including TST and IGRA conversion and downstream development of symptomatic, infectious TB. [62,62]

#### Symptoms or signs

Establishing the presence of symptoms or signs of TB is a trade-off between sensitivity (identifying as many individuals with potential TB) and specificity (avoiding over diagnosing this dimension) [63], as highlighted by the complementary use of CXR in TB prevalence surveys. Widely used screening tools such as presence of cough or the WHO 4-symptom screen are known to omit certain symptoms or signs that people may report if asked. [26,64] Even when present, a symptom or sign may not be reported by the individual, highlighting the difference between subclinical and asymptomatic (Table 2). [64] The optimal approach to assessing TB symptoms or signs will also depend on setting (research, screening programme, clinical care) and goal in terms of sensitivity and specificity.

### Research priorities

Key research priorities were identified during discussions and further developed during topic-specific workshops. Priority areas include diagnosis, interventions for treatment and prevention, defining the individual (morbidity and mortality) and population (transmission and incidence) benefits of intervening during subclinical and non-infectious clinical TB states, and challenges for programmatic implementation (Table 4).

Such research efforts will help with the further operationalisation of the disease dimensions (macroscopic pathology, infectiousness and symptoms or signs), including establishing diagnostic thresholds and where possible quantifying the non-linearity (how quickly individuals move in and out of disease states), as well as enable broader consultations to establish language that can be used in clinical and public interactions.

#### Diagnostics

Key priorities are development of 1) reference standards and 2) validated operational tests for all dimensions of infection and disease. This includes evaluation of existing tools against the new states, as well as the development of new tools. The area of greatest diagnostic need is biomarkers for non-infectious subclinical and clinical TB to increase diagnostic confidence that radiographically evident disease is caused by *Mtb*, in absence of sputum/biopsy microbiological positivity. In addition, while current immunoreactivity tests remain in use despite their limitations, developing a test to confirm *Mtb* infection by demonstrating presence of viable bacilli is key.

#### Treatment

It is likely that the combination of duration and composition for a curative regimen for subclinical TB will lie between that of current TPT for *Mtb* infection and treatment for ‘active TB’ (clinical infectious TB in our framework). A key research priority is therefore to identify the optimal combination, dosage and duration of anti-mycobacterial drugs, as well as any relevant host directed therapies, to effectively treat each TB state and prevent future progression (Figure 2b).

#### Individual benefits

Two priorities were agreed upon: determining 1) the benefit of subclinical TB treatment for reducing TB mortality, recurrence/relapse and post-TB sequelae; and 2) the potential impact of chronic subclinical TB-associated inflammation on exacerbation of comorbidities, including HIV-1, diabetes, lung cancer, cardiovascular and chronic kidney disease. [65,66] This will be aided by a better understanding of the macroscopic and microscopic cellular alterations that correlate with the presentation of subclinical TB.

#### Transmission

The relative infectiousness of infectious-subclinical TB and tools to define infectiousness are key research priorities to determine the benefit of subclinical TB treatment and prevention on community transmission and, consequently, TB incidence.

#### Implementation

Operational and implementation research will need to complement diagnostic development and clinical trials for subclinical TB states and non-infectious clinical pulmonary TB to avoid poorly implemented algorithms and misclassification of individuals resulting in inappropriate treatments. Requirements for adoption include engagement with policymakers, updating of national and international guidelines, training curricula and surveillance systems, as well as engaging with individuals and communities to co-develop acceptable diagnostic and treatment approaches targeted towards each TB state.

## Discussion

The inadequacies of the binary active/latent TB paradigm have been highlighted for several years. There has been increasing recognition, reflected by numerous articles and commentaries, on the need for additional states without a clear strategy on how to move this agenda forward. Here we have taken the next step, bringing together a diverse group of stakeholders and experts to identify the most useful classification that reflects our current understanding of TB conceptual states and relevant research priorities.

The new classification highlights variability in three central dimensions of TB (macroscopic pathology, infectiousness and symptoms or signs), and their separate consideration should result in greater flexibility and accuracy of categorization of a disease that operates on a spectrum. We also highlight how infectiousness can be independent of symptoms, yet disease cannot exist without pathology.

Emphasising how disease pathology can occur without symptoms and infectiousness (the latter as suggested by the detection of *Mtb* in respiratory samples) we provide a potential approach for early diagnosis and intervention in those with subclinical non-infectious TB to prevent progression to infectious TB, thus distinguishing it from TPT for *Mtb* infection.

By definition, the classification is a simplification of the disease process and reflects current understanding and evidence, and as such limitations and compromises are inevitable. Arguments could be made to further subdivide severe or late disease. The framework does not cover all disease scenarios (for example severe disease that may have different prognosis, or post-TB outcome) as its initial purpose is to enable research towards improving TB elimination and therefore, we focused on better defining early disease states. While this consensus group will not have included all voices, by involving a large and diverse group of stakeholders we hope that it reflects a wide range of views to enable broad acceptance.

The process of developing and implementing a new classification will take time and should be revisited at regular intervals to (1) continue to include other views and perspectives, (2) reflect on feedback of groups seeking to implement it in a range of settings, (3) potentially extend the framework, for example to consider advanced disease and post-TB complications and, crucially, (4) incorporate new research findings and diagnostic developments. Areas of emerging research that could influence our understanding of TB are the study of *Mtb* in bioaerosols, investigation of cellular reservoirs of *Mtb*, impact of subclinical inflammation on co-morbidities and post-TB sequela, assessment of viable *Mtb* post-treatment and existence of *Mtb* colonization.

Our proposed classification is conceptual, but the intention is for it to ultimately inform public health and clinical practice as well. Some elements will be immediately relevant to all areas, particularly the awareness of subclinical disease states. By providing a shared framework for the required research, individuals working across fundamental science, qualitative and quantitative approaches, implementation science, and policy research, our framework can have the assurance that results will have broad application, and work towards the required policy shifts. For example, as the binary paradigm led to a binary treatment approach and a one-size fits all approach to treat disease, our classification suggests a reconsideration of existing boundaries between preventive and curative treatment policies. [67] These and other urgent areas of research, including but not restricted to those outlined in the previous section, will need to be taken up over the coming years.

Realising the full potential of the new international consensus classification will require promotion by a broad group of stakeholders, with funding to support key research questions and regular review to revise the conceptual model as necessary. In time the new framework should contribute to TB elimination if it facilitates early diagnosis and effective treatment that optimises patient outcomes and minimises *Mtb* transmission within affected communities.

## Supplementary Material

Appendix

## Figures and Tables

**Table 1:** Participant demographics of those in the online Delphi process and in-person meeting

Participant demographics		Total (n=71)	Delphi (n=51)	Meeting (n=63)
Gender	Female	29 (40.8%)	20 (39.2%)	24 (38.1%)
	Male	42 (59.2%)	31 (60.8%)	39 (61.9%)
Career Stage	Early	11 (15.5%)	3 (5.9%)	10 (15.9%)
	Mid/Late	60 (84.5%)	48 (94.1%)	53 (84.1%)
Nationality	LMIC	39 (54.9%)	26 (51.0%)	32 (50.8%)
	HIC	32 (45.1%)	25 (49.0%)	31 (49.2%)
Region	AFR	22 (31.0%)	10 (19.6%)	22 (34.9%)
	AMR	13 (18.3%)	11 (21.6%)	13 (20.6%)
	EUR	17 (23.9%)	12 (23.5%)	16 (25.4%)
	EMR	2 (2.8%)	2 (3.9%)	2 (3.2%)
	SEAR	10 (14.1%)	9 (17.6%)	5 (7.9%)
	WPR	7 (9.9%)	7 (13.7%)	5 (7.9%)
Stakeholder group	Academic	32 (45.1%)	25 (49.0%)	28 (44.4%)
	Clinical Academic/Clinical Practice	18 (25.4%)	16 (31.4%)	16 (25.4%)
	Patient perspective/Lived experience	3 (4.2%)	2 (3.9%)	3 (4.8%)
	Policy	9 (12.7%)	6 (11.8%)	7 (11.1%)
	Funder	8 (11.3%)	2 (3.9%)	8 (12.7%)
	Industry	1 (1.4%)	0 (0.0%)	1 (1.6%)

**Table 2:** Glossary of terms

Term	Concise definition	Detailed Definition
Viable *Mtb*	Live *Mtb* is present	*Mtb* is present inside the body, and if isolated would grow if cultured appropriately, can cause infected individuals to develop TB without re-exposure to *Mtb*.
Immunoreactivity	Presence of immune memory to current or previous *Mtb* infection	Evidence of an acquired immune response to *Mtb* antigens, typically through skin test or interferon gamma release assays, suggesting that the person has had an infection with *Mtb* at some point either currently or in the past. Response may persist after sterilisation of infection. In the context of immunocompromise the immune response to infection may insufficient to be detected thus being falsely negative.
Host response	Infection or disease specific host response	The host response to *Mtb,* which is multifaceted and evolves through the infection and disease course. A host response can be protective or contribute to disease and influences the outcome of infection.
*Mtb* infection	Viable *Mtb* is present without macroscopic pathology	Viable *Mtb* and an associated host response is present without macroscopic pathology (no disease). The individual has no symptoms or signs consistent with TB and is non-infectious.
TB state	Current presentation of TB	Four disease states are defined by the present combination of three disease dimensions: macroscopic pathology, infectiousness, TB symptoms or signs. Not defined by anticipated future trajectory. The minimum threshold for disease is the presence of macroscopic pathology. Viable *Mtb* and an associated host response is present in each state. The non-disease state of *Mtb* infection lacks all three disease dimension.
Macroscopic Pathology	TB pathology visible with the naked eye, imaging, or tissue examination	The visible manifestation of *Mtb* not being effectively controlled by the host immune response with evidence of cellular infiltration, tissue invasion or destruction. Distinct from a contained granuloma or completely healed lesions, May require high resolution imaging to detect (e.g. CT, PET/CT, MRI).
Infectiousness	The potential ability to cause new *Mtb* infections	An individual is infectious if they aerosolise or expectorate *Mtb* from the respiratory tract which has the potential to cause new infections. Infectiousness is a function of multiple factors, including viability, load and phenotype of the *Mtb.*
Transmission	A new *Mtb* (re-) infection occurs in another host	Transmission occurs when the presence of an infectious individual is coupled with a receptive new host as well as appropriate contact intensity and environment.
Symptoms or signs	Symptoms and signs of TB	Symptoms or signs of TB that are identified through medical history or physical examination.
Subclinical	Individuals are without, not aware of, or not reporting symptoms or signs of TB	Individuals are without, not aware of, or do not report any symptoms during a symptom screen or medical history, and no physical signs that would be recognised as indicative of TB upon clinical examination.
Asymptomatic	Individuals do not have symptoms or signs of TB	Individuals do not have symptoms or signs caused by or related to TB
Diagnostic reference standard	Current best individual/combin ed set of tools to diagnose a disease state	Current best individual or combined set of tools to diagnose a disease state and to assess the accuracy of newer tests. The accuracy of newer tests is expressed as a proportion of reference standard positive or negative.

**Table 3:** Examples of existing diagnostics to identify different disease dimensions

	Disease Dimensions		
Tool Application	Macroscopic Pathology	Infectiousness	Symptoms and Signs
**Potential for use as or incorporation into a reference standard**	Advanced imaging PET/CT CT MRI Histopathology Examination of anatomical samples	BAL culture Induced sputum culture CASS	In-depth symptom interview and clinical exam Objective symptoms evaluation
**Potential for operational use**	Digital CXR +/- CAD	Spontaneous sputum culture	WHO 4 symptom screen Symptom severity scores Cough (≥ 2 weeks or any duration)
**Unclear implications**	Blood or urine tests (eg blood transcriptional markers, serum CRP, urine Mtb antigen detection (ie LAM))*	Spontaneous sputum *Mtb* DNA PCR only Upper respiratory (e.g. mouth) tract swab Bio-aerosol sampling (e.g. face mask sampling)	

Ag, antigen; BAL, bronchoalveolar lavage; CRP, C-Reactive Protein; CXR, chest X-ray; CAD, Computer-Aided Diagnosis; CT, computed tomography; LAM, lipoarabinomannan; MRI, Magnetic resonance imaging; PET/CT, Positron emission tomography/CT; Xpert, GeneXpert MTB/RIF; CASS, Cough Aerosol Sampling System; WHO 4 symptom screen, any one of current cough, fever, night sweats, or weight loss.

* Note, host response related to evident macroscopic pathology could be detected through a validated blood test. This is yet to be determined for existing tests.

**Table 4:** Research priorities and challenges for investigating subclinical and non-infectious TB

Research Gap	Notes/challenges
**Diagnosis**	
Reference standards for new TB states	Validated reference standards need to be developed for subclinical TB and *Mtb* infection.
Validated operational tests for viable *Mtb*	No current tools exist to confirm the presence of *Mtb* in the body when routine microbiological tests are negative (e.g. on sputum for pulmonary TB or biopsies for extrapulmonary TB).
Validated operational tests for host response	A confirmatory biomarker is needed to confirm whether pathology that is evident on imaging is due to a currently active disease process. It is possible that that some biomarkers will be unique across states, reflective of current disease processes (i.e. blood RNA), and some will be detectable across multiple states (i.e. *Mtb*-specific T cell activation).
Validated operational tests for macroscopic pathology	Digital CXR and associated CAD technologies can detect evidence of macroscopic pathology but not with the sensitivity of more high-resolution cross-sectional imaging such as CT which are not practical operational tools. Developments in CXR technology or improvement in AI-based CAD software are needed to facilitate improved detection of macroscopic pathology. No imaging modality i specific for TB pathology hence radiotracers or additional biomarkers should be developed to detect to provide this specificity. Ultimately a diagnostic test capturing the host response associated with macroscopic pathology may omi the need for imaging.
Validated operational tests for infectiousness	Need to establish the performance of new testing modalities (e.g. bio-aerosol, tongue swab) in detecting and quantifying the degree to which index cases can cause new *Mtb* infections, as compared to current sputum-based tests.
Standardised protocol to detect symptoms & signs of TB	Limitations of current thresholds (coush ≥ 2 weeks, WHO 4 symptom screen [any of current cough, fever, night sweats, or weight loss]) are known, but need empirically informed alternatives that provide workable balance between sensitivity and specificity. Need to consider which symptoms and signs to include, their duration and severity.
TB screening strategies	To use tools to measure all three dimensions of disease and report the tests used, to determine sensitivity of approach. Targeting high risk populations and piggybacking or embedding subclinical and non-infectious TB screening activities into other activities can improve TB detection strategies.
**Treatment and prevention**	
Optimal treatment for infectious/non-infectious subclinical TB and non-infectious clinical TB	Determining sufficiency of treatment for non-infectious TB.
Develop research networks that could undertake randomised trials in TB case finding	It is necessary to screen large numbers of individuals to identify new cases, so large multi-site approaches, with shared protocols, may potentially be more efficient.
Prevention of disease	Trials of vaccination to prevent disease should attempt to rule out subclinical TB at enrolment. The benefit/harm of vaccination is unknown if an individual has subclinical TB. Expanding vaccine trial outcomes to earlier states (i.e. beyond infectious clinical disease) may reduce time and costs of clinical studies.
**Individual benefit from treating subclinical TB states**	
Impact on mortality and recurrence by subclinical TB treatment	Opportunities exist for the retrospective analysis of existing cohort data where people in subclinical TB states have received treatment.
Impact on post-TB sequelae by subclinical TB treatment	Evaluation of impact on post-TB sequalae requires multi-modal measurements embedded within subclinical TB treatment ttrials and follow up beyond the end of treatment.
Impact on psychosocial and economic quality of life by subclinical TB treatment	Engagement is needed to develop patient-informed outcomes that assess both desirable and undesirable outcomes.
Impact on comorbidity exacerbation and incidence by subclinical TB treatment	Timing and frequency of measurements needs to be defined by each clinically meaningful outcome. Opportunity for design of new trials for subclinical treatment to include collection of samples/data to assess these measures.
**Population benefit from diagnosing and treating subclinical TB states**	
The potential and benefits of detection and treatment of subclinical TB to reduce transmission and incidence	Historical and contemporary case finding studies have suggested substantial impact on TB prevalence. But studies with clear disease phenotypes are key to provide direct empirical evidence.
Relative infectiousness of subclinical TB infectious states	Requires the identification of phenotypes in a defined population, with subsequent comparison of relative infectiousness metrics.
Tools to measure transmission	It is not possible to ‘prove’ transmission events, immunoreactivity is poorly understood, and new (blood-) based biomarkers of *Mtb* infection would be extremely welcome. These are likely to be challenging to develop.
**Implementation Gaps**	
Programmatic implementation of sub-disease management	Implementation will require sufficient diagnostics, treatments and algorithms to be developed to avoid misclassification and inappropriate treatment
Translating new framework into practice	Requires significant changes to recording and reporting tools. Modification to guidelines, training and surveillance systems.
Cost-effectiveness of subclinical and non-infectious TB detection	To inform the likely economic benefits to TB program budgets, cost effectiveness evaluations will need to be conducted. The balance between resource-intensive mass screening initiatives and short-long term benefits to impact on clinical management costs needs to be weighed up.
Implications of population-based screening on individuals	Individuals and communities need to be engaged to evaluate potential stigma and implementation challenges, as well as benefits of integration in existing community initiatives and health screening programs.

## Key messages

- **Need** - There is a need for clarity and consistency in the terminology used for conceptual tuberculosis (TB) states and criteria to enable progress in research towards improvements in clinical practice and policy.
- **Process** - We achieved consensus through a Delphi process with wide representation from geographical locations, gender, professional disciplines, as well as lived and working experiences
- **Disease dimensions** - Three disease dimensions were considered to define TB states 1) macroscopic pathology, 2) infectiousness and 3) symptoms & signs.
- **Disease Threshold** - Defined as the presence of macroscopic pathology irrespective of infectiousness or symptoms and signs
- **Disease framework** - Four disease states were defined: non-infectious and infectious subclinical TB, as well as non-infectious and infectious clinical TB.
- **Non-linearity** - After pathology develops, people may fluctuate between infectious and non-infectious states, and between the presence and absence of symptoms or signs.
- **Diagnostic tools** - We have imperfect tools to accurately identify the various conceptual disease states. Developing better tools to facilitate research using the conceptual framework is an urgent priority.
- **Flexibility** - The ICE-TB framework will be responsive to new evidence and insight, with adaptation as needed.
