# Supplementary material for: International consensus classification of early tuberculosis states to guide research for improved care and prevention: A Delphi exercise
Source: Lancet Respir Med. Author manuscript; Available in PMC 2024 Jul 31. (PMC7616323; doi:10.1016/S2213-2600(24)00028-6)
Supplement: Appendix [file EMS197676-supplement-Appendix.pdf]

Appendix

International consensus classification of early tuberculosis states to guide research for improved care and prevention: A Delphi exercise

Anna K Coussens (PhD) #, Syed MA Zaidi (MPH), Brian A Allwood (PhD), Puneet K Dewan (MD), Prof Glenda Gray (MD), Mikashmi Kohli (PhD), Tamara Kredo (PhD), Prof Ben J Marais (PhD), Prof Guy Marks (PhD), Leo Martinez (PhD), Morten Ruhwald (PhD), Prof Thomas J Scriba (PhD), Prof James A Seddon (PhD), Phumeza Tisile (BA), Prof Digby F Warner (PhD), Prof Robert J Wilkinson (PhD), Hanif Esmail (PhD)*#, Prof Rein MGJ Houben (PhD)# **on behalf of the International Consensus for Early TB (ICE-TB) group**

*Corresponding author
#Contributed equally

Table of Contents

Appendix 1 - Consensus process methodology 2
Appendix 2 - Meeting participants 4
Appendix 3 - Online Delphi survey results 15
Appendix 4 - Terminology 30
Appendix 5 - In-person meeting agenda 32

Appendix 1 - Consensus process methodology

Consensus process

Delphi process - online surveys

The main purpose of two online surveys (see below for details) was to explore areas of agreement and disagreement within the group to inform discussions at the in-person meeting. The first survey explored the groups perspectives on TB states, pathophysiology, natural history and the need for a novel framework and terminology. Results then informed the second survey, which included more focused questions on the key steps in early pathogenesis ofTB, and the conceptual features for a disease state.

Questions for the two surveys were developed and piloted by the SOC with further feedback from the SC. Participants were given 2-3 weeks to complete each survey independently. It was highlighted that the presentation of the results would be anonymous and that responses should not be shared with others to minimise social desirability biases. Most questions required responses on a five-point Likert scale (1.Strongly disagree, 2. Disagree, 3. Neither agree nor disagree, 4. Agree, 5. Strongly Agree) with the opportunity for detailed comments. Formal criteria for consensus were not used during this stage as the purpose was to explore areas of agreement and disagreement within the group to inform the subsequent in-person meeting.

First Online Delphi survey

The first survey explored perspectives within the group on TB states, pathophysiology, natural history (including the dimensions that define disease and what should be considered disease), and the need for a novel framework and terminology.

Second Online Delphi survey

Results from the first Delphi survey informed the second, which included more focused questions on the key steps in early TB pathogenesis, and the conceptual features for a disease state. .

Delphi process - in-person meeting

The in-person meeting consisted of presentations, workshops, panel and small group discussions and consensus generating activities. Presentations focused on presenting results from the scoping review as well as key areas of agreement and disagreement from the online Delphi surveys. Two sets of four parallel workshops consisted of smaller subgroups of 14-16 participants. The first set of workshops covered key disciplines/areas in

TB (bacteriology and transmission; imaging; immunology; public health, modelling and epidemiology; extrapulmonary, and paediatric disease) to discuss key issues that arose from plenary discussions. The second set focused on research gaps, in particular the benefits and challenges for programmatic implementation of the new disease framework (see Appendix 5 for a full meeting agenda). The panel discussions were an opportunity for key stakeholders to reflect and expand on topics discussed in the meeting and to debate issues of controversy.

The in-person meeting consisted of presentations, workshops, panel and small group discussions and consensus generating activities. Key plenary consensus activities included the entire consortium and were moderated by an expert impartial methodologist (TK), with experience in chairing consensus meetings and guideline development but from outside the TB field hence providing impartiality. Ground rules were outlined at the beginning and included respectful interaction, where differences of opinion were taken as helpful opportunities to explore diverse views. Several polls were held throughout the meeting to determine the degree of consensus for each part of the framework.

Attendees were given the opportunity to be voting or non-voting participants in the consensus building process with several (n=7) individuals from e.g. funding organisations contributing as non-voting members. Participants were provided with green and red cards to express either agreement or disagreement. It was emphasised that agreement could encompass views ranging from full agreement to a “can live with” a statement, whereas disagreement represented a fundamental disagreement and a desire wanting to block a statement. Where there was disagreement within the group the aim was to resolve this by discussion but if disagreement remained significant after several rounds of discussion a formal vote was made with an agreed threshold of 70% of voting participants to indicate consensus.

Despite the challenging content and many areas of uncertainty in the underlying evidence, polls indicated that the consensus threshold was exceeded for all key statements reached, therefore no formal votes were required. We captured key considerations and potential reasons for dissent. The full meeting agenda is provided below.

Appendix 2 - Meeting participants

Participant List						
Name	Affiliation and country	Role	Stakeholder Group	Delphi participant	Meeting participant	ICE-TB Group Member
Adam Penn-Nicholson	TB programme, FIND, Switzerland	Invited expert	Policy	No	Yes	Yes
Adrie JC Steyn	Department of Microbiology and Centers for AIDS Research and Free Radical Biology, University of Alabama at Birmingham, AL, USA; Africa Health Research Institute, University of KwaZulu Natal, South Africa	Invited expert	Academic	Yes	Yes	Yes
Alvaro Schwalb	Instituto de Medicina Tropical Alexander von Humboldt, Peru; Infectious Disease Epidemiology, London School of Hygiene and Tropical Medicine, UK	Invited expert	Clinical Academic/ Clinical Practice	Yes	Yes	Yes
Andrew A Vernon	National Institute of Allergy and Infectious Diseases, NIH, USA	Invited expert	Funder	Yes	Yes	No
Ann Ginsberg	Tuberculosis, Bill & Melinda Gates Foundation, USA	Invited expert	Funder	No	Yes	No
Anna K Coussens	Infectious Diseases and Immune Defence Division, The Walter and Eliza Hall Institute (WEHI), Australia; Centre for Infectious Diseases Research in Africa, Institute of Infectious Disease and Molecular	SOC	Academic	Yes	Yes	Yes

	Medicine, University of Cape Town, South Africa; Department of Medical Biology, University of Melbourne, Australia					
Ben J Marais	Sydney Infectious Diseases Institute (Sydney ID) and the WHO Collaborating Centre in Tuberculosis	Invited expert	Clinical Academic/ Clinical Practice	Yes	Yes	Yes
Brian W Allwood	Division of Pulmonology, Department of Medicine, Stellenbosch University & Tygerberg Hospital, South Africa;	Invited expert	Clinical Academic/ Clinical Practice	Yes	Yes	Yes
Busisiwe B Beko	TB Proof, South Africa	Invited expert	Patients and lived experience	Yes	Yes	Yes
C Padmapriyadarsini	ICMR - National Institute for Research in Tuberculosis, India	Invited expert	Clinical Academic/ Clinical Practice	Yes	No	No
Caroline ML Williams	Department of Respiratory Sciences, University of Leicester, UK	Invited expert	Clinical Academic/ Clinical Practice	Yes	Yes	Yes
Cecily R Miller	Global TB Programme, WHO, Switzerland	Invited expert	Policy	Yes	No	No
Charlotte L Weller	Wellcome Trust, UK	Invited expert observer	Funder	No	Yes	No
David Alland	Public Health Research Institute, New Jersey Medical School, Rutgers University, USA.	Invited expert	Academic	Yes	Yes	Yes
Dharanidharan Ramamurthy	Molecular Mycobacteriology Research Unit, Institute of Infectious Disease and	ECR Rapporteur	Academic	No	Yes	Yes

	Molecular Medicine, University of Cape Town, South Africa					
Digby Warner	Department of Pathology, University of Cape Town; Molecular Mycobacteriology Research Unit and Centre for Infectious Diseases Research in Africa, Institute of Infectious Disease and Molecular Medicine, University of Cape Town, South Africa	SOC	Academic	Yes	Yes	Yes
Divya K Shah	Wellcome Trust, UK	Invited expert observer	Funder	No	Yes	No
Donald Simon	DSI-NRF Centre of Excellence for Biomedical Tuberculosis Research, South African Medical Research Council Centre for Tuberculosis Research, Division of Molecular Biology and Human Genetics, Biomedical Research Institute, Faculty of Medicine and Health Sciences, Stellenbosch University, South Africa	ECR Rapporteur	Clinical Academic/ Clinical Practice	No	Yes	Yes
Dylan Sheerin	Infectious Diseases and Immune Defence Division, The Walter and Eliza Hall Institute (WEHI), Australia; Centre for Infectious Diseases Research in Africa, Institute of Infectious Disease and Molecular Medicine, University of Cape Town, South Africa;	ECR Rapporteur	Academic	No	Yes	Yes

	Department of Medical Biology, University of Melbourne, Australia					
Elisa Nemes	South African Tuberculosis Vaccine Initiative, Institute of Infectious Disease and Molecular Medicine and Division of Immunology, Department of Pathology, University of Cape Town, South Africa	Invited expert	Academic	Yes	Yes	Yes
Emily A Kendall	Center for Tuberculosis Research, Division of Infectious Diseases, Johns Hopkins University, USA	Invited expert	Clinical Academic/ Clinical Practice	Yes	Yes	Yes
Emily B Wong	Africa Health Research Institute, University of KwaZulu Natal, South Africa; Division of Infectious Diseases, Department of Medicine, Heersink School of Medicine, University of Alabama, USA	Invited expert	Clinical Academic/ Clinical Practice	Yes	Yes	Yes
Erlina Burhan	Persahabatan Hospital/Department of Pulmonary and Respiratory Medicine, Faculty of Medicine, Universitas Indonesia, Indonesia	Invited expert	Clinical Academic/ Clinical Practice	Yes	Yes	Yes
Frank Cobelens	Amsterdam University Medical Centers location University of Amsterdam, Netherlands	Invited expert	Academic	Yes	Yes	Yes
Gaurang Tanna	Bill & Melinda Gates Foundation, TB Delivery, South Africa	Invited expert observer	Funder	No	Yes	No

Gavin Churchyard	Aurum Institute, South Africa	Invited expert	Academic	Yes	Yes	Yes
Gerhard Walzl	DSI-NRF Centre of Excellence for Biomedical Tuberculosis Research, South African Medical Research Council Centre for Tuberculosis Research, Division of Molecular Biology and Human Genetics, Biomedical Research Institute, Faculty of Medicine and Health Sciences, Stellenbosch University, South Africa	Invited expert	Academic	Yes	Yes	Yes
Glenda E Gray	South African Medical Research Council, South Africa	SC	Policy	No	Yes	Yes
Guy B Marks	Department of Clinical Medicine, Faculty of Medicine and Health, University of NSW, Australia	SC	Clinical Academic/ Clinical Practice	Yes	Yes	Yes
Hai Viet Nguyen	Vietnam National TB Program, Vietnam	Invited expert	Academic	Yes	No	No
Hanif Esmail	MRC Clinical Trials Unit at University College London; Insititute for Global Health, University College London, UK; Centre for Infectious Diseases Research in Africa, Insitute of Infectious Diseases and Molecular Medicine, University of Cape Town, South Africa	SOC	Academic	Yes	Yes	Yes
James A Seddon	Department of Infectious Disease, Imperial College London, London, United Kingdom AND	SOC	Clinical Academic/ Clinical Practice	Yes	Yes	Yes

	Desmond Tutu TB Centre, Department of Paediatrics and Child Health, Stellenbosch University, South Africa					
Jerrold J Ellner	Division of Infectious Diseases, Center for Emerging Pathogens, New Jersey Medical School, Rutgers University, USA	Invited expert	Academic	Yes	Yes	Yes
Jingtao Gao	Clinical Center on TB, Beijing Chest Hospital, Capital Medical University, China.	Invited expert	Policy	Yes	No	No
Justin T Denholm	Victorian Tuberculosis Program, Melbourne Health; Department of Infectious Diseases, University of Melbourne	Invited expert	Clinical Academic/ Clinical Practice	Yes	Yes	Yes
Kate A Haigh	Centre for Infectious Diseases Research in Africa, Institute of Infectious Disease and Molecular Medicine; Institute of Infection, Veterinary and Ecological Sciences	ECR Rapporteur	Clinical Academic/ Clinical Practice	No	Yes	Yes
Katherine C Horton	Department of Infectious Disease Epidemiology and Dynamics	Invited expert	Academic	Yes	Yes	Yes
Leonardo Martinez	Department of Epidemiology, Boston University School of Public Health, USA	Invited expert	Academic	Yes	Yes	Yes
Marcel A Behr	Department of Medicine, McGIll University	Invited expert	Clinical Academic/ Clinical Practice	Yes	Yes	Yes

Mark Hatherill	South African Tuberculosis Vaccine Initiative, Institute of Infectious Disease and Molecular Medicine and Division of Immunology, Department of Pathology, University of Cape Town, South Africa	Invited expert	Academic	Yes	Yes	Yes
Mikashmi Kohli	Health Programmes, FIND, Switzerland	SC	Policy	Yes	Yes	Yes
Molebogeng X Rangaka	MRC Clinical Trials Unit at University College London; Insititute for Global Health, University College London, UK; Centre for Infectious Diseases Research in Africa, Institute of Infectious Disease and Molecular Medicine, University of Cape Town, South Africa	Invited expert	Academic	Yes	Yes	Yes
Morten Ruhwald	TB programme, FIND, Switzerland	SC	Academic	Yes	Yes	Yes
Munyaradzi Musvosi	South African Tuberculosis Vaccine Initiative, Institute of Infectious Disease and Molecular Medicine and Division of Immunology, Department of Pathology, University of Cape Town, South Africa	ECR Rapporteur	Academic	No	Yes	Yes
Nazir A Ismail	Global TB Programme, WHO, Switzerland	SC	Policy	Yes	Yes	No
Nguyen Thu Anh	Faculty of Medicine and Health, Woolcock Institute of Medical Research, University of Sydney	Invited expert	Academic	Yes	Yes	Yes

Nim Arinaminpathy	Medical Research Council Centre for Global Infectious Disease Analysis, Imperial College London, UK	Invited expert	Academic	Yes	No	No
Padmini Salgame	Division of Infectious Diseases, Center for Emerging Pathogens, New Jersey Medical School, Rutgers University, USA	Invited expert	Academic	Yes	No	No
Palwasha Y Khan	Data Science Unit, Africa Health Research Institute, South Africa ; Department of Clinical Research, London School of Hygiene and Tropical Medicine, UK	Invited expert	Clinical Academic/ Clinical Practice	Yes	Yes	Yes
Peter Kim	National Institute of Allergy and Infectious Diseases, NIH, USA	SC	Funder	No	Yes	No
Peter MacPherson	School of Health & Wellbeing, University of Glasgow, UK; Malawi-Liverpool-Wellcome Programme, Malawi, and London School of Hygiene & Tropical Medicine, UK	Invited expert	Clinical Academic/ Clinical Practice	Yes	Yes	Yes
Phumeza Tisile	TB proof, South Africa	SC	Patients and lived experience	No	Yes	Yes
Pren Naidoo	Bill & Melinda Gates Foundation, TB Delivery, South Africa	Invited expert observer	Funder	No	Yes	No
Puneet K Dewan	Tuberculosis & HIV, Bill & Melinda Gates Foundation, USA	SC	Funder	Yes	Yes	Yes
Razia Fatima	The common management unit to manage TB HIV AIDS and Malaria, Pakistan	Invited expert	Policy	Yes	Yes	Yes

Rein MGJ Houben	Infectious Disease Epidemiology, London School of Hygiene and Tropical Medicine, UK	SOC	Academic	Yes	Yes	Yes
Robert J Wilkinson	Centre for Infectious Diseases Research in Africa, Institute of Infectious Disease and Molecular Medicine, University of Cape Town, South Africa; Imperial College London, UK; Francis Crick Institute, UK	SC	Clinical Academic/ Clinical Practice	Yes	Yes	Yes
Robin Wood	Institute of Infectious Disease and Molecular Medicine, University of Cape Town, South Africa; Desmond Tutu Health Foundation, South Africa	Invited expert	Academic	No	Yes	Yes
Roxana Rustomjee	BioNTech, USA	Invited expert observer	Industry	No	Yes	No
Ryan Dinkele	Molecular Mycobacteriology Research Unit, Institute of Infectious Disease and Molecular Medicine, University of Cape Town, South Africa	ECR Rapporteur	Academic	No	Yes	Yes
Sandip Mandal	Independent Consultant, India	Invited expert	Academic	Yes	No	No
Sayera Banu	Emerging Infections Program, Infectious Diseases Division, ICDDR, Bangladesh	Invited expert	Clinical Academic/ Clinical Practice	Yes	No	No
Simon C Mendelsohn	South African Tuberculosis Vaccine Initiative, Institute of Infectious Disease and Molecular Medicine and Division of Immunology,	ECR Rapporteur	Academic	No	Yes	Yes

	Department of Pathology, University of Cape Town, South Africa					
Siyan Yi	School of Public Health, National Institute of Public Health, Phnom Penh, Cambodia; Saw Swee Hock School of Public Health, National University of Singapore and National University Health System, Singapore; KHANA Center for Population Health Research, Cambodia; Center for Global Health Research, Touro University California, USA	Invited expert	Policy	Yes	Yes	Yes
Stephanus T Malherbe	DSI-NRF Centre of Excellence for Biomedical Tuberculosis Research, South African Medical Research Council Centre for Tuberculosis Research, Division of Molecular Biology and Human Genetics, Biomedical Research Institute, Faculty of Medicine and Health Sciences, Stellenbosch University, South Africa	Invited expert	Academic	No	Yes	Yes
Suvanand Sahu	Stop TB Partnerhip, Switzerland	Invited expert	Patients and lived experience	Yes	Yes	Yes
Syed MA Zaidi	Institute for Global Health, University College London, National University of Medical Sciences, Pakistan	SOC	Academic	Yes	Yes	Yes

Tamara Kredo	Health Systems Research Unit, South Africa Medical Research Council, Cape Town, South Africa	SOC	Policy and methodology	No	Yes	Yes
Thomas J Scriba	South African Tuberculosis Vaccine Initiative, Institute of Infectious Disease and Molecular Medicine and Division of Immunology, Department of Pathology, University of Cape Town, South Africa	Invited expert	Academic	Yes	Yes	Yes
Vidya Mave	Johns Hopkins Center for Infectious Diseases in India, Pune, India	Invited expert	Academic	Yes	Yes	Yes
Yingda L Xie	Division of Infectious Diseases, Center for Emerging Pathogens, New Jersey Medical School, Rutgers University, USA	Invited expert	Academic	Yes	Yes	Yes

Participant selection and list

A list of potential participants was drafted by the Scientific Organising Committee (SOC, with further input by the Steering Committee (SC). Invitations were sent to expert nominees from the list to best reflect representation from geographical locations with balance in income settings, gender, professional disciplines and working experiences until 44 experts accepted, giving a total of 60 participants including the SOC (7) and SC (9). The 60 participants were invited to complete the Delphi surveys, of which 54 accepted and 6 indicated they would be an observer for the surveys. Three expert nominees withdrew from participation after agreeing to participate due to schedule conflict. Eight participants in the Delphi surveys did not attend the in-person meeting due to delays in visa approval (n=6) and scheduling conflict (n=2). Invitations were sent to a further 3 experts (2 accepted) and 5 expert observers (5 accepted). Seven Early Career Researchers were invited from local universities through an open call to act as observers and rapporteurs.

Appendix 3 - Online Delphi survey results

Delphi 1:

Histograms all reflect the number of participants selecting each option

Section 1: Adequacy of Binary Paradigm

Responses to Question: A binary paradigm of latent TB (infection) and active TB (disease) is sufficient to inform research for global TB elimination

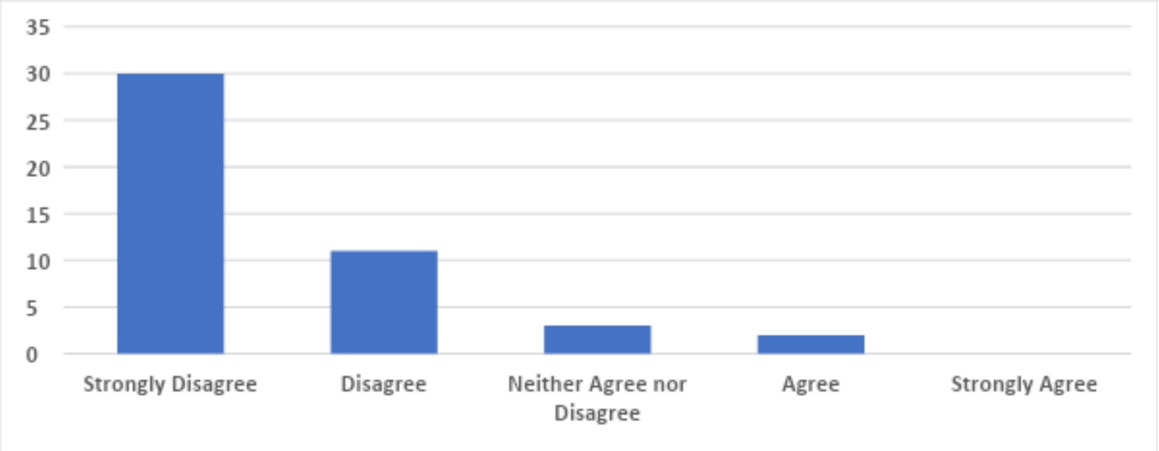

Responses to Question: A binary paradigm of latent TB (infection) and active TB (disease) is sufficient programmatically for TB elimination.

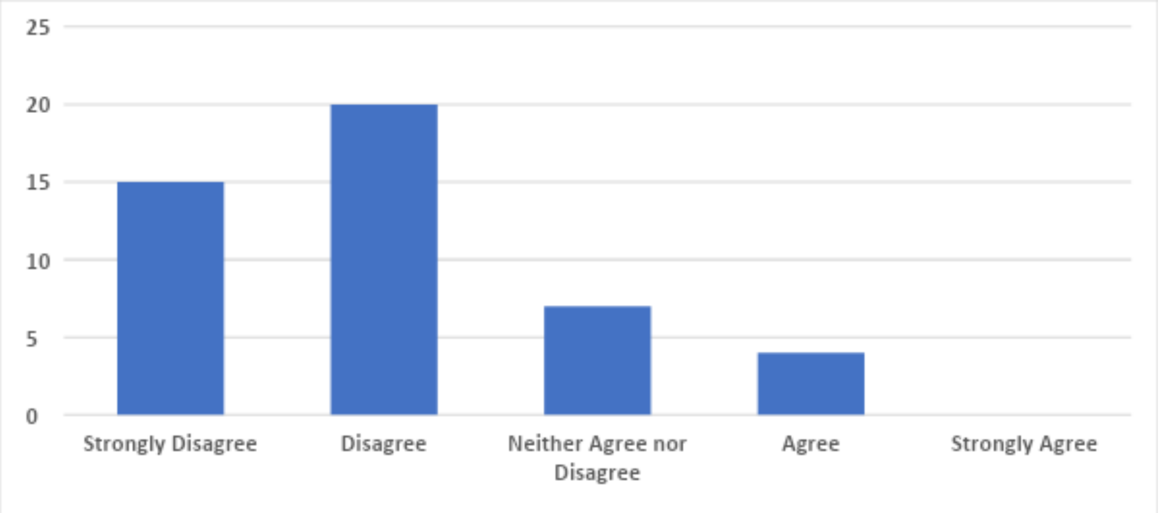

Responses to Question: It would be useful to apply multiple (more than two) stages for TB, such as, is carried out for cancer.

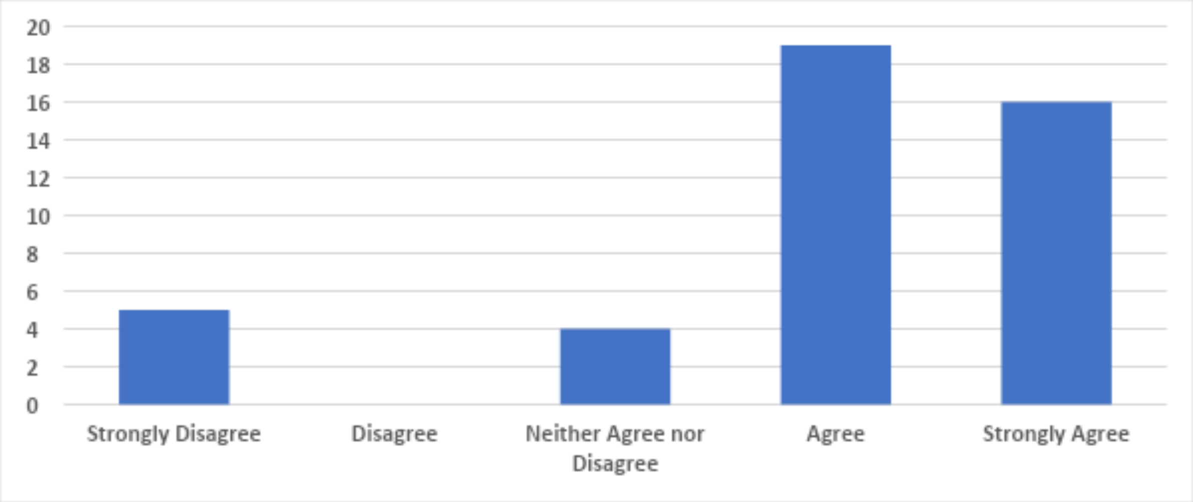

Qualitative Responses

Common themes to question: In what ways is a binary paradigm sufficient or insufficient for research?

- Over-simplification
- Does not capture complexity of disease
- Limits understanding of transmission
- Excludes subclinical disease

"The binary paradigm is a hopeless simplification of a complex interaction."

Common themes to question: In what ways is a binary paradigm sufficient or insufficient for programs?

- Early / asymptomatic / subclinical/ intermediate stages may contribute to transmission.
- Simplification is a missed opportunity.
- Diagnostics and treatment are challenging / unclear.

"Programs need straightforward and simple terminology and procedures to manage nationwide care."

Section 2: TB Pathogenesis

Factors that should be utilized to determine a particular stage - ranked highest to lowest *(Participants were asked to rate the factors mentioned below, Maximum Score =10)*

Factor	Mean Score	SD
Transmission potential / infectiousness	8.6	2.0
Ability to discriminate using current or future diagnostics	8.4	1.9
Potential approaches to current or future treatment (e.g, duration / regimen)	7.8	2.5
Prognostic differences between states	7.6	1.9
Extent of pathology and tissue damage	6.5	2.7
Bacillary load	5.9	2.8
Health-seeking behavior	4.9	2.8

Responses to Question: Please mark all points where the individual should be considered as having TB disease?

(Participants were asked to rate each stage mentioned below, Maximum Score =5)

Pathogenesis Stage	Mean Score	SD
Point at which Mtb is first taken up by a host cell (e.g. alveolar macrophage)	1.3	0.7
Point at which a T cell memory or antibody response to Mtb is generated	1.5	0.9
Point at which a granuloma is formed containing replicating Mtb	2.4	1.3
Point at which the individual is not able to self-eradicate Mtb despite generating an acquired immune response (i.e., Mtb persists with the host)	2.8	1.4
Point at which inflammatory/infiltrative pathology to Mtb is evident through imaging	3.9	1.0
Point at which Mtb can be detected from sputum	4.4	0.9
Point at which an individual develops TB symptoms	4.6	0.9
Point at which an individual seeks healthcare	4.0	1.4

Qualitative Responses

Common themes to question: Please describe in a few words what the term “TB disease” means to you

- Pathology and tissue damage
- Signs and symptoms

• Requiring treatment

“When Mtb has caused pathological damage, i.e. healthy tissue has stopped functioning due to Mtb”

Responses to Question: A single approach to TB staging must apply to both pulmonary and extra-pulmonary TB

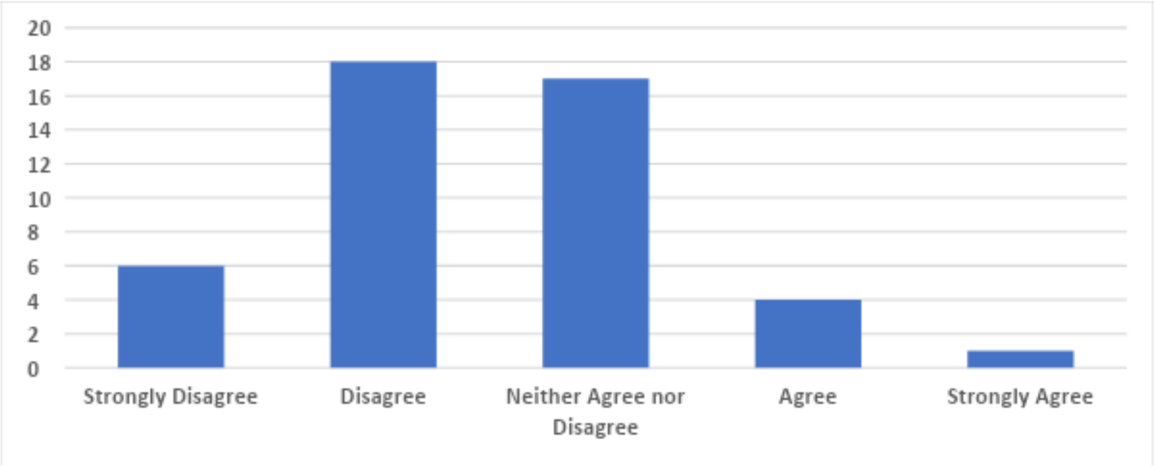

Responses to Question: A single approach to TB staging must apply to both young children and adolescents / adults

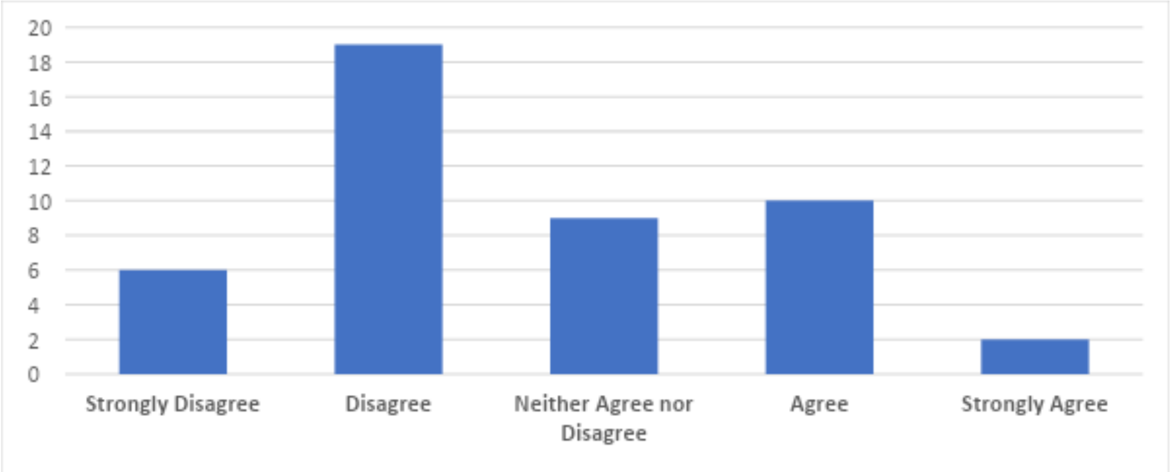

Qualitative Responses

Common themes to question: Why have you stated this opinion?

• Heterogeneous

• Clinical presentation is diverse and disease trajectory are different.

- No opinion / unsure

"It would be great if we had a single approach, but given the divergent manifestation of childhood and adult TB disease phenotypes, this is likely unrealistic."

"Extrapulmonary TB will generally (if not, always) not be infectious, so it might be difficult to fix a universal definition."

Section 3: Terminology

Most popular terms to describe different TB stages- ranked highest to lowest

Term	Mean Score	SD
Pulmonary TB	4.4	0.9
Extra-pulmonary TB	4.4	0.9
Disseminated TB	4.1	1.3
Subclinical TB	4.0	1.2
Bacteriologically positive TB disease	3.8	1.4
Previous History of TB Treatment	3.7	1.2
Symptomatic TB	3.7	1.3
Cavitary TB	3.7	1.4
Mtb infection	3.6	1.5
Post-TB	3.4	1.4
Clinical TB	3.2	1.5
Mtb immune sensitization	3.2	1.5
Asymptomatic Disease	3.1	1.3
Bacteriologically negative TB disease	3.0	1.4
Active TB	3.0	1.5
Paucibacillary TB	2.9	1.4
TB infection	2.9	1.6
Previous TB	2.9	1.5
Symptom-screen negative TB	2.8	1.6
Incipient TB	2.8	1.4
Severe TB	2.7	1.4
Early TB	2.7	1.4
Previous TB History	2.7	1.5
Advanced TB	2.6	1.4

Minimal TB	2.6	1.4
Latent TB	2.4	1.4
Primary TB	2.3	1.4
Asymptomatic infection	2.3	1.4
Past TB	2.1	1.2
Post-primary TB	2.0	1.3
Non-severe TB	2.0	1.2
Quiescent TB	1.8	1.1
Inactive TB	1.7	1.0
Dormant TB	1.6	0.9
Percolating TB	1.5	0.9

(Participants were asked to rate each term mentioned below, Maximum Score =5)

Section 4: Research Priorities

Qualitative Responses

Common themes for research priorities listed by participants.

- Infectiousness of early / subclinical / people without symptoms
- Sensitivity / use of CXR and AI software for detection of early stages
- Biomarkers for prediction / early detection / identify disease
- Better tools for detecting EP and childhood TB
- Shorter, simpler, safer regimens for treatment
- Strategies for mass-screening, cost-effectiveness and use of X-rays
- Reduction in post-TB, improved outcomes through early treatment

Delphi 2:

Section 1: Key relevant steps in early TB disease pathogenesis

1. Granuloma(s) can fail to control *Mtb*; this results in further spread of *Mtb*, and causes a host-derived cellular infiltration within or into the surrounding tissue which can become macroscopically evident and initially occur without development of symptoms.

Responses to Question: To what extent do you agree or disagree that this is a key step in TB pathogenesis.

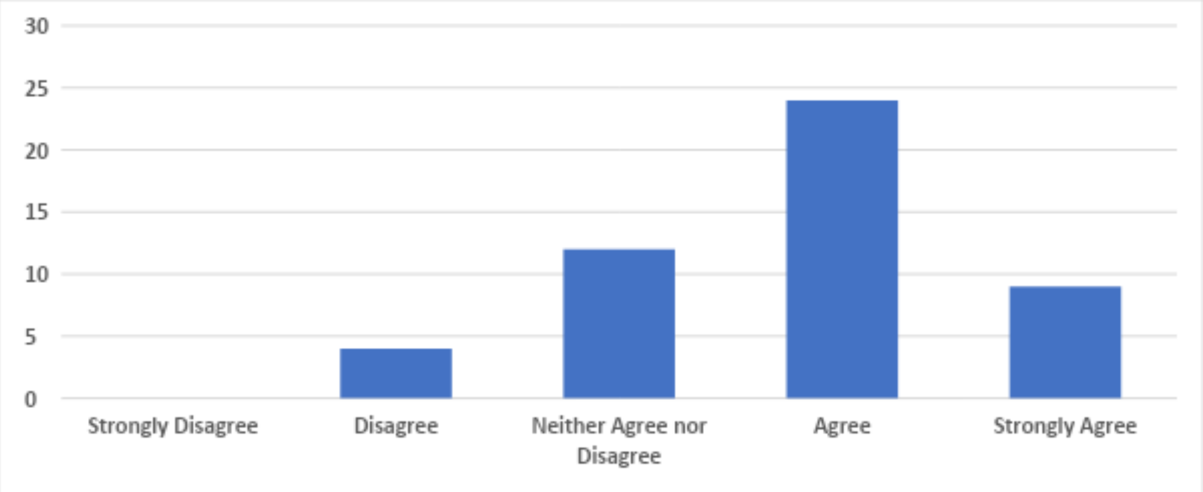

2. As Mtb replicates and spreads locally, the host generates a characteristic immune response that is distinguishable from the response to (many) other pathogens

Responses to Question: To what extent do you agree or disagree that this is a key step in TB pathogenesis.

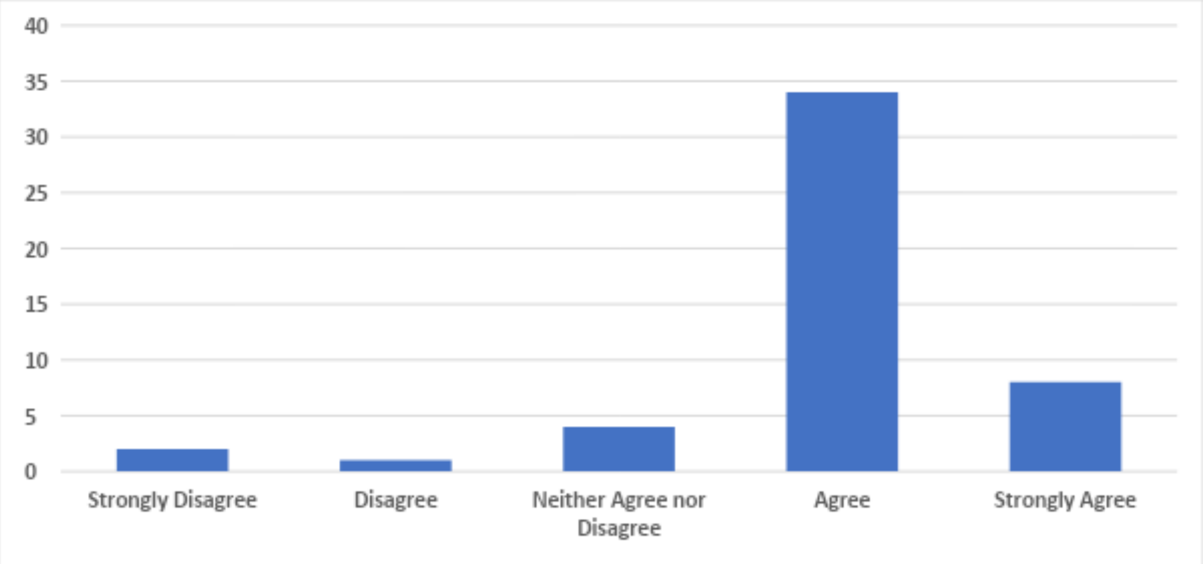

3. Within the lungs, as *Mtb* replicates and spreads, bacilli can be shed into airways resulting in aerosolization, facilitating transmission.

Responses to Question: To what extent do you agree or disagree that this is a key step in TB pathogenesis.

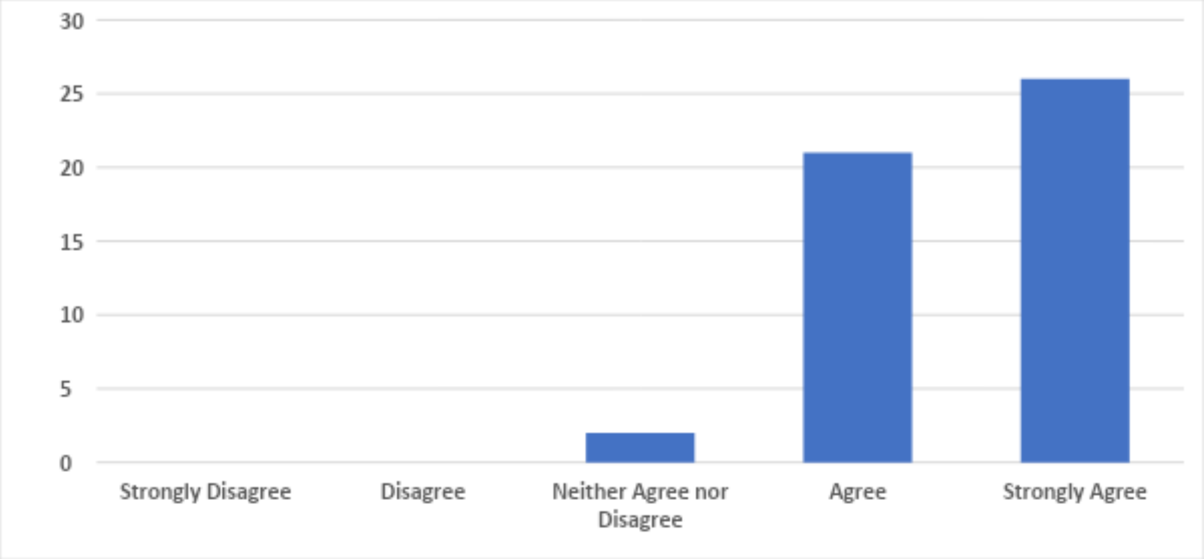

4. As *Mtb* replicates and spreads locally, the host immune response and associated anatomical disruption can lead to symptoms.

Responses to Question: To what extent do you agree or disagree that this is a key step in TB pathogenesis.

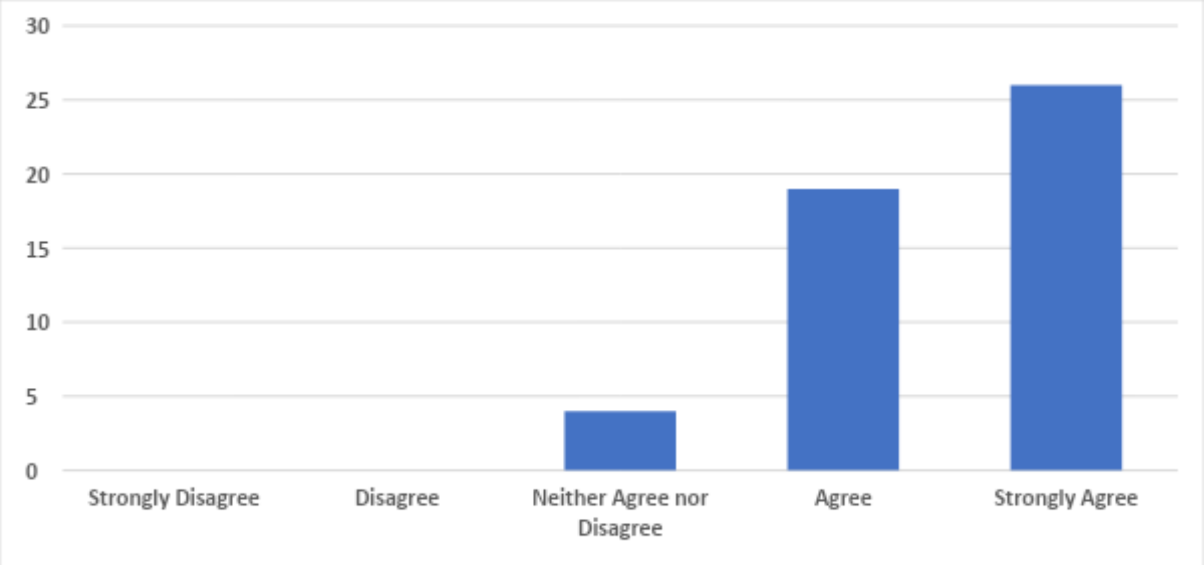

Qualitative Responses

Common themes to question: Are there any other relevant steps to early TB disease pathogenesis that you think should be highlighted?

- Colonization, initial local spread, primary progression
- Hematogenous spread/ non-pulmonary
- Non-linearity / reversibility

“This is all about local spread in the lungs. It is possible that following one of the two early stages, there is dissemination without either spread of bacilli or symptoms that might lead to seeding to other sites. I think this could be a stage?”

Section 2: Overall conceptual framework for TB staging

Stage 1 - Features consistent with this proposed conceptual stage:

- No symptoms related to TB or, if present, not sufficient to seek care
- No presence of macroscopically evident pathology related to TB disease (i.e. No disease pathology that would be visible to the naked eye)
- Mtb-specific immune response detectable in blood or through skin testing
- No viable Mtb in respiratory secretions or aerosols - hence non-infectious via the respiratory route
- Potential for progression in the future to a stage that has positive respiratory secretions or aerosols - hence to become infectious via the respiratory route
- Can be adequately treated (progression prevented) by therapy for “latent” TB

Responses to Question: Do you agree this is conceptually the current definition of “latent” TB

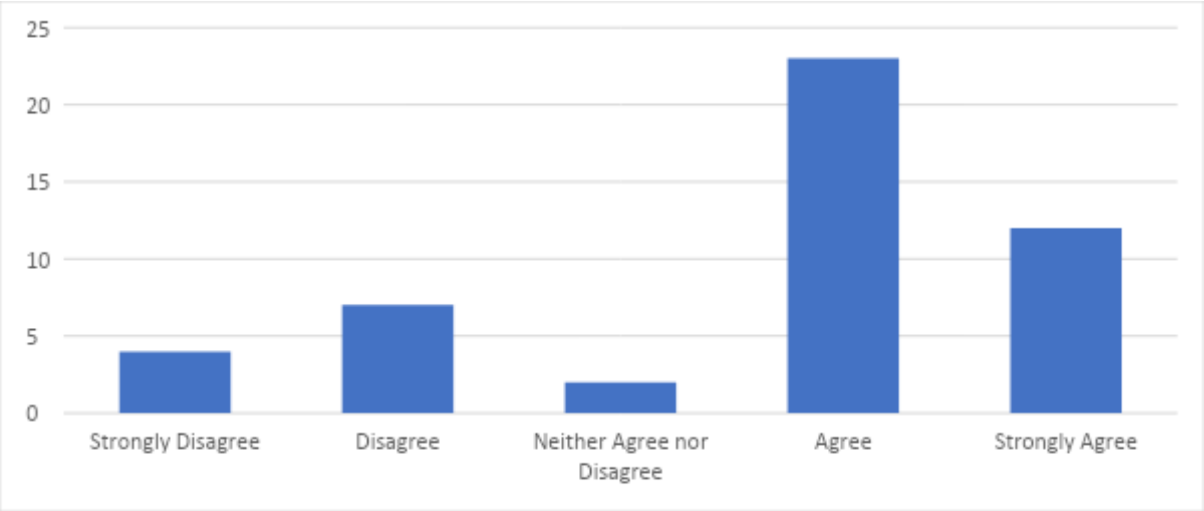

Stage 2 - Features consistent with this proposed conceptual stage:

- No symptoms related to TB or if present not sufficient to seek care
- Presence of macroscopic pathology related to TB (i.e. disease pathology that would be visible to the naked eye)
- No viable Mtb in respiratory secretions or aerosols - hence non-infectious via the respiratory route
- Potential for progression in the future to a stage that has positive respiratory secretions or aerosols - hence to become infectious via the respiratory route
- Not adequately treated by therapy for “latent” TB
- Potential to be distinguished from those without TB disease by an Mtb-specific immune response, TB stage-specific biomarker signature or Mtb antigen/Mtb detection in blood, other bodily fluid or tissue samples.

Responses to Question: To what extent do you agree or disagree that this is conceptually an important stage of TB disease to distinguish that if diagnosed could provide benefit to the individual, society or further enable progress towards TB elimination?

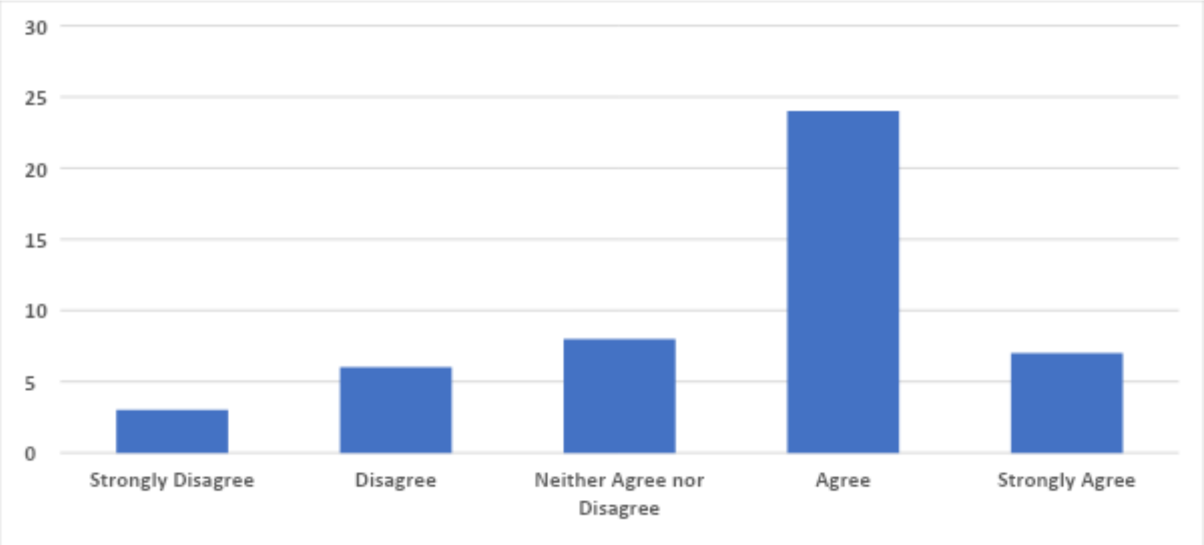

Stage 3 - Features consistent with this proposed Stage:

- No symptoms related to TB or if present not sufficient to seek care
- Presence of macroscopic pathology related to TB (i.e. disease pathology that would be visible to the naked eye)
- Viable Mtb in respiratory secretions or aerosols - hence infectious via the respiratory route
- Not adequately treated by therapy for “latent” TB

Responses to Question: To what extent do you agree or disagree that this is conceptually an important stage of TB disease to distinguish that if diagnosed could provide benefit to the individual, society or further enable progress towards TB elimination.

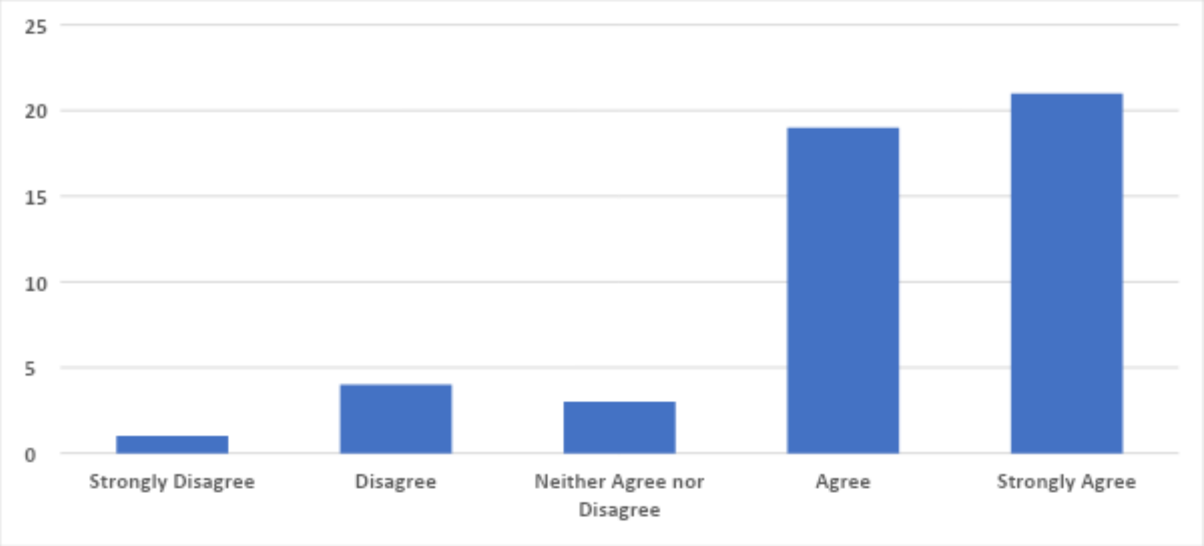

Stage 4 - Features consistent with this proposed Stage:

- Symptoms related to TB sufficient to seek care
- Presence of macroscopic pathology related to TB (i.e. disease pathology that would be visible to the naked eye)
- No viable Mtb in respiratory secretions or aerosols - hence non-infectious via the respiratory route
- Potential for clinical deterioration and progression in the future to a stage that has positive respiratory secretions or aerosols - hence to become infectious via the respiratory route
- Not adequately treated by therapy for “latent” TB
- Potential to be distinguished from those without TB disease by an Mtb-specific immune response, TB-specific biomarker signature or Mtb antigen/Mtb detection in blood, other bodily fluid or tissue samples.

Responses to Question: To what extent do you agree or disagree that this is conceptually an important stage of TB disease to distinguish that if diagnosed could provide benefit to the individual, society or further enable progress towards TB elimination.

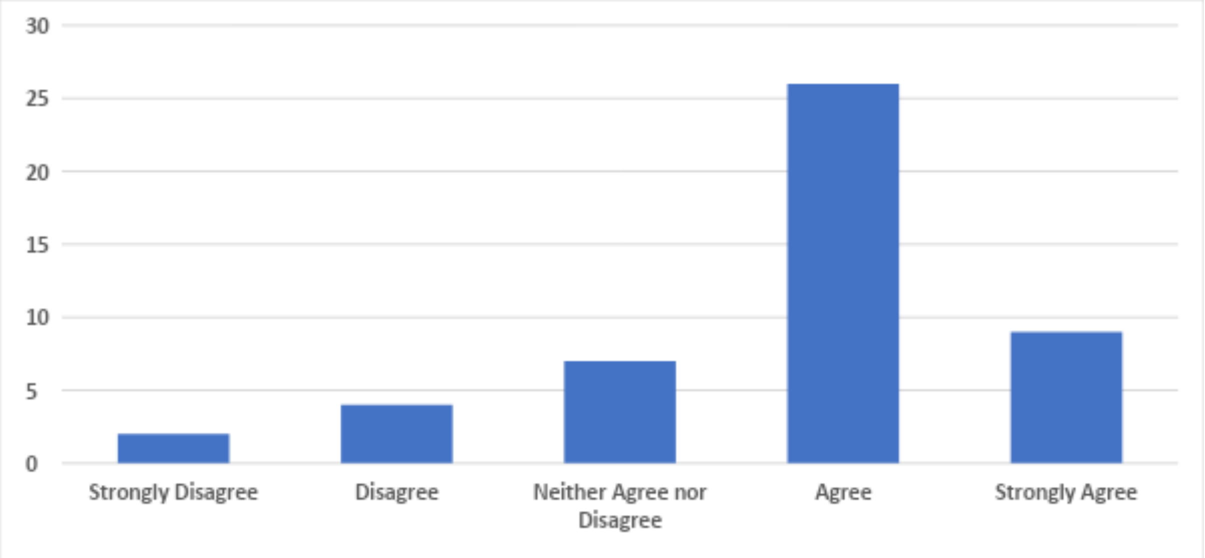

Stage 5 - Features consistent with this proposed Stage:

- Symptoms related to TB sufficient to seek care
- Presence of macroscopically evident pathology related to TB disease
- Viable Mtb in respiratory secretions or aerosols - hence infectious via the respiratory route

Response to Question: Do you agree this is conceptually the current definition of “active” TB

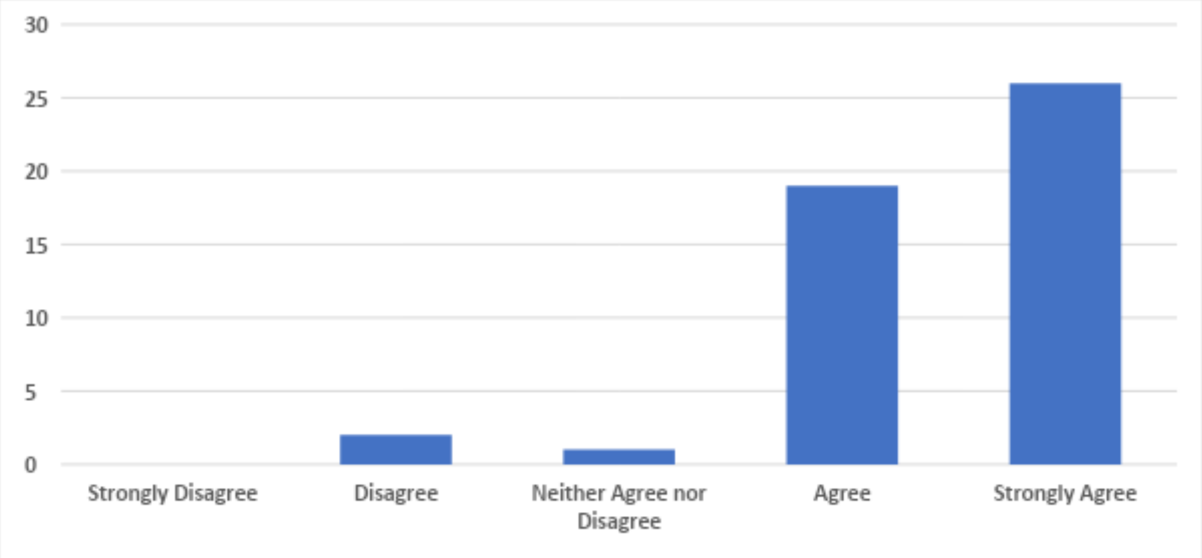

Part 3: Criteria for development of diagnostic staging

Participants were asked whether they agreed, disagreed (or neither) to the below statements

	**Statement**	**% Agreeing**
1	Our aim should be to have a single TB staging system that is implementable in low- and high- resource settings,	75.6%
2	Our aim should be to have a single TB staging system that is applicable for clinical practice, policy and research (*i.e.*, different staging systems in different settings should be avoided)	73.3%
3	A TB stage must be diagnosable using currently routinely used or widely available diagnostic tests (*e.g.,* sputum Xpert, Chest X-Ray).	28.9%
4	A TB stage may be diagnosable using diagnostic tests that are not widely available (*e.g.*, induced sputum culture, CT scan)	55.6%
5	A TB stage may be diagnosable using research biomarkers / diagnostic tests in development. (*e.g.*, transcriptional signatures, bioaerosol sampling)	64.4%

Qualitative Responses to Diagnostic Criteria

- New tools, more time, data & resources for research before diagnostics can be rolled out
- Clarity needed on purpose of staging: clinical, research or individual risk prediction
- Diagnostics availability should not be limiting factor
- Diagnostics under development should not be considered
- Need to set goals to develop diagnostics

"I think the staging system should drive the development of diagnostics, not the other way around."

"The ultimate aim must be to have widely available diagnostics in all regions, but there must be scope for a period of time where we could define these stages based on tests with research/limited availability, then move to roll out more widely."

"The development and use of new tests and insight should be encouraged, but ideally with reference to readily available test results to provide clinical context."

Diagnostic Criteria

The following diagnostic criteria were listed for TB stages in the survey.

Responses to Question: To what extent to you agree or disagree that these criteria are useful and adequately capture conceptual stages

**Stage**	***Antigen Response*** **(IGRA/TST)**	***Radiology*** **(CXR)**	***Bacteriology*** **(Spontaneous Sputum Xpert/Culture)**	***Symptoms*** **(Symptom screen)**	**% Agreeing**
1	Positive	Negative	Negative	Negative	64.4%
2	Positive or Negative	Positive	Negative/ Unobtainable	Negative	46.7%
3	Positive or Negative	Positive or Negative	Positive	Negative	75.6%

4	Positive or Negative	Positive	Negative	Positive	44.4%
5	Positive or Negative	Positive	Positive	Positive	88.9%

Participants were asked to list additional tests that can be used in the diagnostic criteria - common themes are listed below.

Stage	Additional diagnostic tests that should be included or developed
1	Tests for antigen-specific T-cells, Diagnostics for viable / replicating MTB infection, Microbial biomarkers, New generation skin tests
2	Activation of Mtb-specific T cells, Blood Mtb (e.g. cfDNA/Mtb peptides in extracellular vesicles), RNA signatures, CAD, HRCT, Repat CXRs
3	Mask or tidal breath sampling, Volatile compounds diagnostics, LAM or other biomarkers that detect the organism (DNA/RNA, antigens, phenotypic expression)
4	Oral swabs - additional cultures, induced sputum, Pathogen or immune signatures for clinically significant disease, Tests that distinguish this from non-TB pathogens as causes, HIV status, Aerosol release
5	Blood based tests for TB disease, POC tests or self-test LAM, RNA signatures, sputum free testing, Prognostic biomarker - including response to treatment.

Participants were asked to for their preferred terms for each state – most common terms are listed below

Stage	Preferred Terms for Stage
1	TB infection, Mtb infection, Latent TB,
2	Minimal TB, Early TB,
3	Subclinical TB, Asymptomatic TB
4	Clinical TB, Bacteriologically negative TB
5	Active TB, Symptomatic TB

Appendix 4 - Terminology

Terminology – general considerations

The approach to terminology was extensively explored in the online Delphi process and in-person meeting. As shown by Zaidi *et al*., [21] many terms have been used to describe various TB states, with overlap in places. Consensus was reached on several choices, while recognising that terminology is inherently contentious and no choice will meet complete support. A key principle was that terminology should be as clear and unambiguous as possible.

First, we posit that tuberculosis (TB) ‘is’ a disease and that using the expression ‘tuberculosis disease’ is repetitive, as if to say ‘cancer disease’. This is consistent with the origin of the term relating to the presence of tubercles as the common pathology in the disease. [70] Secondly, to better reflect the agreed non-linearity of TB, there was consensus to use the term ‘state’, rather than ‘stage’, as the latter would suggest a temporary situation from which (linear) progression is expected.

Another key discussion point was the use of terms subclinical/clinical and asymptomatic/symptomatic TB, both of which refer to (the absence of) symptoms and signs of TB and have been used widely. [21] The group agreed that subclinical described individuals without, not aware of, or not reporting symptoms or signs of TB, whereas asymptomatic was defined as individuals not experiencing any symptoms and without signs. While this definition of subclinical makes the actual threshold time- and place dependent, a more definitive term, such as asymptomatic, was considered to be potentially misleading or impractical, in particular for a concept as inherently subjective as symptoms and signs.

There was agreement to include the term infectious (see Table 1 for definition) as part of state descriptions, given the importance of transmission potential for clinical management of patient and contacts, public health implications and policy impact measurement.

Incipient TB

The term "incipient TB” has gained popularity and was the subject of discussion during the consensus process. As part of the scoping review it was noted the term “incipient” itself has been in use since the early 19th century and formed part of the initial 1917 National Tuberculosis Association (NTA) classification of TB and then re-emerged recently. While its definition has evolved, it still broadly captures the concept of very early disease with expected progression. Notably the term was dropped by the NTA as the non-linear

framework of TB natural history in this classification meant that people could return to the state of incipient TB after many years of progression, which was felt illogical. .

Recently “incipient” TB was defined in a WHO/FIND Target Product Profile as “*Individuals with tuberculosis infection in whom progression to TB disease has started and who have no symptoms, no radiographic abnormalities suggestive of TB and negative microbiological investigations. Individuals with incipient disease are very likely to develop active TB within a short time of initial evaluation. A subset of patients with incipient disease (primarily immunocompetent patients) will not progress to active disease*”. The sensitivity of radiographic approach is not defined here, with development in ultra-high resolution CT the spatial resolution of medical imaging can be <0.25mm providing a very limited window for this state where disease has started to progress but is not visible radiographically. The absence of radiographic abnormalities is at odds with more historic use of the term where the condition was predicated on the presence of radiographic abnormalities. In addition further description of incipient TB in the WHO/FIND TPP, states that incipient TB may include periods of healing and disease regression as evidenced by radiographic and pathological findings which is internally inconsistent. Hence in practice what is considered as incipient TB by this definition will be captured within our Subclinical Non-infectious state. Moreover incipient TB is conceptually defined as having two outcomes (progression or regression) yet TPP diagnostic evaluation was only assessed against one outcome (developing TB within 2 years). Hence the term intends to capture a transition between states predicated on only one future outcome, and not the independent current state. For these reasons the consensus was to not includ it in the framework

Appendix 5 - In-person meeting agenda

1st International Symposium on
New Concepts in Early TB Disease

1-2 FEBRUARY 2023
THE VINEYARD HOTEL
NEWLANDS, CAPE TOWN | SOUTH AFRICA

PROGRAMME DAY 1 | 1 FEB

Time	Session	Chairs
07:00–08:00	**BREAKFAST FOR HOTEL GUESTS**	
08:00–08:30	**REGISTRATION AND BREAKFAST FRUIT & PASTRIES**<br>**Vineyard Conference Centre – Level 2 Camphor**	
08:30–09:30	**SESSION 1: OPENING SESSION**<br><br>**Objectives**<br>• Share objectives, clarify scope of meeting<br>• Present results of the scoping review<br><br>**Opening:** Robert Wilkinson, Peter Kim, Puneet Dewan, Phumeza Tisile (15 mins)<br><br>**Scope of meeting:** Hanif Esmail (10 mins)<br><br>**Introductions, expectations, process:** Tamara Kredo (25 mins)<br><br>**Results of scoping review on Early TB:** Asad Zaidi (10 mins)	**Anna Coussens, Rein Houben**
09.30–10.30	**SESSION 2: CURRENT STATE OF CONSENSUS & KEY ISSUES**<br><br>**Objectives**<br>• Present results from two-stage Delphi process<br>• Explore key issues/controversies<br>• Progressing discussion on key areas for consensus<br><br>**Report back on Delphi process:** Asad Zaidi (15 mins)<br><br>**Key issues and controversies in early TB** (45 mins)<br>**Introduction by co-chairs**<br>a) **Disease threshold:** *What is the place for biomarkers in the diagnostic criteria for conceptual stages of TB disease?* Anna Coussens<br>b) **Diagnostic criteria:** *What are implications of gold standard (i.e. high sensitivity) versus routine tests on the size and distribution of TB disease states?* Rein Houben<br>c) **Diagnostic criteria:** *Should presence of care-seeking symptoms define a state in individuals with not-infectious TB/absence of Mtb aerosolisation?* Frank Cobelens<br>d) **Framework:** *Should a single TB disease framework cover adult PTB as well as EPTB and Paed TB?* James Seddon	**Guy Marks, Puneet Dewan**

PROGRAMME DAY 1 | 1 FEB

Time	Session	Chairs
10:30–11:00	**SESSION 3: DISEASE STAGING**<br>**Objective**<br>• Progress discussion on key areas for consensus<br>**Discussion on disease staging:** Exploring consensus and divergent views (Tamara Kredo 30 min)	**Hanif Esmail, Tamara Kredo**
11:00–11:30	**TEA BREAK**	
11:30–12:30	**SESSION 3 (cont): DISCUSSION AND PARTICIPANT INPUT**<br>**Discussion on disease staging:** Exploring consensus and divergent views (Tamara Kredo 30 min)	
12:30–13:30	**LUNCH – VINEYARD HOTEL (Morii Dining Room)**	
13:30–14:00	**SESSION 4: PREPARATION FOR WORKSHOPS DAY 1**<br>**Objectives**<br>• Present results consensus discussion<br>• Establish purpose and questions for workshops<br>**Feedback from discussion of consensus statements on disease staging:** (Tamara Kredo, 15 mins)<br>**Outline Day 1 workshops and key questions**<br>(Workshop chairs, 15mins) :	**Anna Coussens, Tamara Kredo**
14:00–16:00 (including break)	**SESSION 5: DAY 1 WORKSHOPS**<br>**Objective**<br>• Address key questions that are preventing consensus<br>1. Bacteriology and transmission potential of early TB<br>2. Imaging of early TB<br>3. Immunology/biomarkers of early TB<br>4. EPTB / Pediatric early TB	**Workshop co-chairs**<br>**Terrace 1 & 2**<br>**Camphor**<br>**Boardroom 1**<br>**Boardroom 2**
16:00–17:30	**SESSION 6: WORKSHOP REPORTING AND CONSENSUS BUILDING**<br>**Objectives**<br>• Share and discuss findings/proposals from Day 1 workshops<br>• Reflect on discussions<br>**Reporting back from workshop** (Rapporteurs 60 mins)<br>**Panel discussion advancing consensus:** Reflections on discussions and implications for their TB activities (30 mins)	**Digby Warner, Tamara Kredo**
18:30	**PRE-DINNER DRINKS**<br>**Vineyard Conference Centre – Summerhouse lawns**	
19:30	**DINNER**<br>**Vineyard Conference Centre – Summerhouse L1**	

Time	Session	Chairs
07:00–08:00	**BREAKFAST FOR HOTEL GUESTS**	
08:00– 09:00	**SESSION 7: RECAP, DIAGNOSTIC CRITERIA & TERMINOLOGY**<br>**Objectives**<br>• Establish key progress from day 1 and remaining intended outcomes<br>• Establish key issues in diagnostic criteria and terminology<br>• Explore consensus on remaining areas<br><br>**Recap day 1 plus remaining areas of non-consensus:** (Tamara Kredo 15mins)<br><br>**Key issues and controversies in early TB** (45 mins)<br>**Introduction by co-chairs**<br>a) **Diagnostic Criteria:** *Can you diagnose a conceptual state of using a non-specific test?* Emily Kendall<br>b) **Diagnostic Criteria:** *Can non-validated tests be part of a diagnostic definition?* Mikashmi Kohli<br>c) **Diagnostic Criteria:** *What are patient priorities for diagnosis and treatment of early TB?* Busi Beko<br>d) **Terminology:** *Defining conceptual status versus diagnostic criteria – how do we avoid mistakes from the past?* Marcel Behr	**Ann Ginsberg, Morten Ruhwald**
09:00–09:30	**SESSION 8: DIAGNOSTIC CRITERIA & TERMINOLOGY**<br>**Objective**<br>• Discuss consensus on remaining areas<br><br>**Discussion on diagnostic criteria and terminology:** consensus and divergent views (Tamara Kredo 30 min)	**James Seddon, Tamara Kredo, Asad Zaidi**
09:30–10:00	**TEA BREAK**	
10:00–12:00	**SESSION 8 (cont): DIAGNOSTIC CRITERIA & TERMINOLOGY**<br>**Discussion on diagnostic criteria and terminology:** Exploring consensus and divergent views (Tamara Kredo 60 min)	
12:00– 13:00	**LUNCH – VINEYARD HOTEL (Morii Dining Room)**	
13:00 –1 4:00	**SESSION 9: PREPARATION FOR WORKSHOPS DAY 2**<br>**Objectives**<br>• Present results consensus discussion<br>• Establish further questions for workshops<br><br>**Remaining areas of non-consensus and questions to resolve:** (Tamara Kredo, 45 mins)	**Digby Warner, Tamara Kredo**

Time	Session	Chairs
	**Outline Day 2 workshops and key questions:** (Workshop chairs, 15mins)	
14:00–16:00 (including break)	**SESSION 10: DAY 2 WORKSHOPS**<br><br>**Objectives**<br>• Formulate key research areas for Early TB<br>• Address any questions regarding consensus statements<br><br>1. Population benefits - contribution to transmission<br>2. Treatment - different treatment regimes, trial designs and outcomes of interest<br>3. Individual benefits - contribution to post-TB and co-morbidity interactions<br>4. Systematic screening and treating - patient provider, policy maker perspective	Workshop co-chairs<br><br>Terrace 1 & 2<br>Boardroom 2<br>Boardroom 1<br>Camphor
16:00–17:45	**SESSION 11: WORKSHOP REPORTING AND NEXT STEPS**<br><br>**Objectives**<br>• Share and discuss proposed research from Day 2 workshops<br>• Provide final list of consensus statements<br>• Establish next steps and close<br><br>**Reporting back from workshops:** (Rapporteurs, 60 mins)<br><br>**Summary of consensus framework and terminology, remaining areas of contention:** (Tamara Kredo, 30 mins)<br><br>**Next steps and close:** (Rein Houben, 15 mins)	Rein Houben, Hanif Esmail

W1.1

Bacteriology and transmission potential of early TB

TERRACE 1 & 2

NAME	ROLE
Digby Warner	Co-Chair/Speaker
Rein Houben	Co-Chair
David Alland	Speaker
Palwasha Khan	Speaker
Robin Wood	
Katherine Horton	
Emily Kendall	
Frank Cobelens	
Puneet Dewan	
Andrew Vernon	
Vidya Mave	
Divya Shah	
Caroline Williams	Rapporteur
Ryan Dinkele	ECR Rapporteur

W1.2

Imaging of early TB

CAMPHOR ROOM

NAME	ROLE
Hanif Esmail	Co-Chair/Speaker
Asad Zaidi	Co-Chair/Speaker
Razia Fatima	
Emily Wong	Speaker
Peter MacPherson	Speaker
Yingda (Linda) Xie	
Guy B. Marks	
Justin Denholm	
Robert J Wilkinson	
Gavin Churchyard	
Adrie JC Steyn	Speaker
Brian Allwood	
Nazir Ismail	
Morten Ruhwald	
Fanie Malherbe	
Donald Simon	ECR Rapporteur

W1.3

Immunology/biomarkers of early TB

BOARDROOM 1

NAME	ROLE
Anna Coussens	Co-Chair/Speaker
Tom Scriba	Co-Chair/Speaker
Adam Penn-Nicholson	Rapporteur/Speaker
Elisa Nemes	Speaker
Gerhard Walzl	Speaker
Alvaro Schwalb	
Ann Ginsberg	
Jerrold Ellner	
Peter Kim	
Erlina Burhan	
Mikashmi Kohli	
Roxana Rustomjee	
Charlie Weller	
Simon Mendelsohn	ECR Rapporteur
Munyaradzi Musvosi	ECR Rapporteur
Dylan Sheerin	ECR Rapporteur

W1.4

EPTB / Pediatric early TB

BOARDROOM 2

NAME	ROLE
James Seddon	Co-Chair
Ben Marais	Co-Chair/Speaker
Leo Martinez	Co-Chair/Speaker
Phumeza Tisile	
Buci Beko	
Nguyen Thu Anh	
Siyan Yi	
Lele Rangaka	
Sahu Suvanand	
Marcel Behr	
Mark Hatherill	
Pren Naido	
Gaurang Tanna	
Dharanidharan Ramamurthy	ECR Rapporteur
Kate Haigh	ECR Rapporteur

W2.1

Population benefits - contribution to transmission

TERRACE 1 & 2

NAME	ROLE
Rein Houben	Co-Chair
Digby Warner	Co-Chair
Frank Cobelens	
Caroline Williams	
Puneet Dewan	
Robin Wood	
Emily Kendall	All Speaking
David Alland	
Ben Marais	
Peter MacPherson	
Palwasha Khan	
Charlie Weller	
Leo Martinez	Rapporteur
Ryan Dinkele	ECR Rapporteur
Dharanidharan "Joe" Ramamurthy	ECR Rapporteur

W2.2

Treatment - different treatment regimes, trials designs and outcomes of interest

BOARDROOM 2

NAME	ROLE
Hanif Esmail	Co-Chair/Speaker
James Seddon	Co-Chair/Speaker
Andrew Vernon	
Mark Hatherill	Speaker
Prof Glenda Gray	
Yingda (Linda) Xie	Speaker
Nazir Ismail	
Robert J Wilkinson	
Gavin Churchyard	
Gerhard Walzl	
Peter Kim	
Divya Shah	
Adam Penn-Nicholson	
Buci Beko	
Simon Mendelsohn	ECR Rapporteur
Dylan Sheerin	ECR Rapporteur

W2.3

Individual benefits - contribution to post-TB and co-morbidity interactions

BOARDROOM 1

NAME	ROLE
Brian Allwood	Co-Chair/Speaker
Anna Coussens	Co-Chair/Speaker
Katherine Horton	Rapporteur/Speaker
Vidya Mave	Speaker
Razia Fatima	Speaker
Emily Wong	Speaker
Adrie JC Steyn	Speaker
Lele Rangaka	Speaker
Jerrold Ellner	Speaker
Tom Scriba	Speaker
Ann Ginsberg	
Marcel Behr	
Fanie Malherbe	
Donald Simon	ECR Rapporteur
Kate Haigh	ECR Rapporteur

W2.4

Systematic screening and treating - patient provider, policy maker perspective

CAMPHOR ROOM

NAME	ROLE
Asad Zaidi	Co-Chair
Phumeza Tisile	Co-Chair
Morten Ruhwald	Speaker
Guy B. Marks	
Nguyen Thu Anh	
Siyan Yi	
Elisa Nemes	
Erlina Burhan	
Mikashmi Kohli	
Alvaro Schwalb	
Sahu Suvanand	
Roxana Rustomjee	
Pren Naido	
Gaurang Tanna	
Justin Denholm	Rapporteur
Munyaradzi Musvosi	ECR Rapporteur
